# Supplementary material for: Bio-Oriented Synthesis and Molecular Docking Studies of 1,2,4-Triazole Based Derivatives as Potential Anti-Cancer Agents against HepG2 Cell Line
Source: Pharmaceuticals (Basel). 2023 Jan 30;16(2):211. doi: 10.3390/ph16020211 (PMC9964635; doi:10.3390/ph16020211)
Supplement: Supplementary file 1 [file pharmaceuticals-16-00211-s001.zip › pharmaceuticals-2077496-supplementary.pdf]

Supplementary Data

Article

Bio-Oriented synthesis and molecular docking studies of 1,2,4-Triazole based derivatives as potential Anticancer agents against HepG2 Cell Line

Naheed Akhter [1], Sidra Batool [2], Samreen Gul Khan [2,*], Nasir Rasool [2], Fozia Anjum [2], Azhar Rasul [3], Şevki Adem [4], Sadaf Mahmood [2], Aziz-ur-Rehman [5], Mehr-un-Nisa [6], Zainib Razzaq[1], Jørn B. Christensen [7], Mohammed A.S Abourehab [8], Syed Adnan Ali Shah [9, 10*], Syahrul Imran [10, 11].

[1] Department of Biochemistry, Faculty of Life Science, Government College University Faisalabad, Faisalabad 38000, Pakistan
[2] Department of Chemistry, Drug Design and Medicinal Chemistry Laboratory, Faculty of Physical Science, Government College University, Faisalabad 38000, Pakistan
[3] Department of Zoology, Faculty of Life Sciences, Government College University Faisalabad, Faisalabad 38000, Pakistan
[4] Department of Chemistry, Faculty of Sciences, Çankırı Karatekin University, 18100 Çankırı, Turkey
[5] Department of Chemistry, Government College University, Lahore 54000, Pakistan
[6] Department of Chemistry, University of Lahore, Lahore 40100, Pakistan
[7] Department of Chemistry, Faculty of Science, University of Copenhagen, 2100 Copenhagen, Denmark
[8] Department of Pharmaceutics College of Pharmacy, Umm Al-Qura University, Makkah 21955, Saudi Arabia
[9] Faculty of Pharmacy, Universiti Teknologi MARA Cawangan Selangor Kampus Puncak Alam, Bandar Puncak Alam 42300, Selangor D. E., Malaysia
[10] Atta-ur-Rahman Institute for Natural Product Discovery (AuRIns), Universiti Teknologi MARA Cawangan Selangor Kampus Puncak Alam, Bandar Puncak Alam 42300, Selangor D. E., Malaysia
[11] Faculty of Applied Sciences, Universiti Teknologi MARA Shah Alam, Shah Alam 40450, Selangor D.E., Malaysia
* Correspondence: samreengul@gcuf.edu (S.G.K.); syedadnan@uitm.edu.my (S.A.A.S.); Tel.: +92-300-427-0077 (S.G.K.); +60-3-3258-4616 or +60-19365-1307 (S.A.A.S.)

Table of Contents:

Figure S1: ^{1}H NMR spectrum of compound **2** (Full spectrum)....3

Figure S2: ^{1}H NMR spectrum of compound **3** (Full spectrum)....3

Figure S3: ^{1}H NMR spectrum of compound **4**(Full spectrum)....4

Figure S4: ^{1}H NMR spectrum of compound **4** (aromatic region)....4

Figure S5: ^{1}H NMR spectrum of compound **4** (aliphatic region)....5

Figure S6: ^{13}C NMR spectrum of compound **2** 5

Figure S7: ^{13}C NMR spectrum of compound **3** 6

Figure S8: ^{13}C NMR spectrum of compound **4** 6

Figure S9: ^{1}H NMR spectrum of compound **7a** 7

Figure S10: COSY- ^{1}H NMR spectrum of compound **7a** 7

Figure S11: ^{13}C NMR spectrum of compound **7a** 8

Figure S12: ^{1}H NMR spectrum of compound **7b** 8

Figure S13: COSY- ^{1}H NMR spectrum of compound **7b**....9

Figure S14: ^{13}C NMR spectrum of compound **7b** 9

Figure S15: ^{1}H NMR spectrum of compound **7c** 10

Figure S16: COSY- ^{1}H NMR spectrum of compound **7c**....10

Figure S17: ^{13}C NMR spectrum of compound **7b** 11

Figure S18: ^{1}H NMR spectrum of compound **7d** 11

Figure S19: COSY ^{1}H NMR spectrum of compound **7d** 12

Figure S20: ^{13}C NMR spectrum of compound **7d** 12

Figure S21: ^{1}H NMR spectrum of compound **7e** 13

Figure S22: COSY ^{1}H NMR spectrum of compound **7e**....13

Figure S23: ^{13}C NMR spectrum of compound **7d** 14

Figure S24: ^{1}H NMR spectrum of compound **7f** 14

Figure S25: COSY ^{1}H NMR spectrum of compound **7f** 15

Figure S26: ^{13}C NMR spectrum of compound **7f** 15

Figure S27: HRMS spectrum of compound **7a**....16

Figure S28: HRMS spectrum of compound **7b**....17

Figure S29: HRMS spectrum of compound **7c**....18

Figure S30: HRMS spectrum of compound **7d**....19

Figure S31: HRMS spectrum of compound **7e**....20

Figure S32: HRMS spectrum of compound **7f**....21

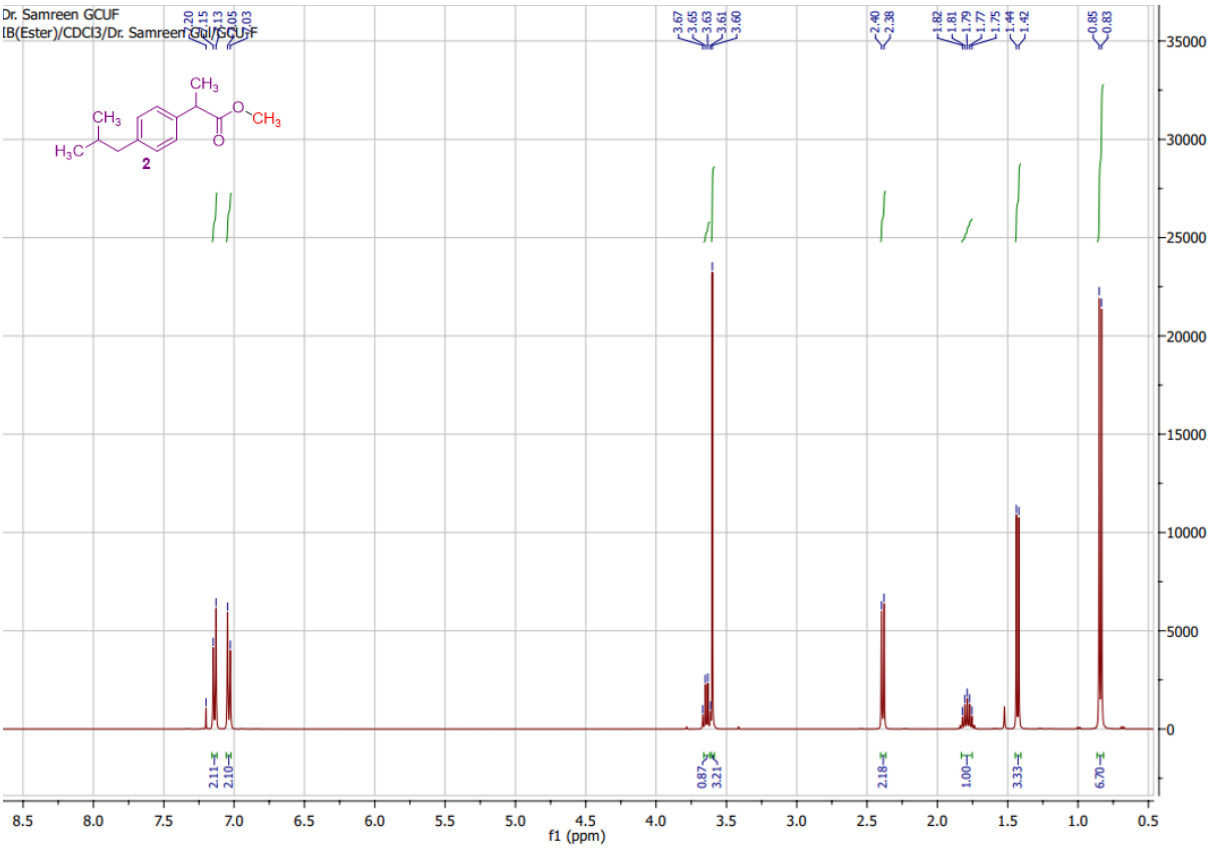

Figure S1: ^{1}H NMR spectrum of compound **2** (Full)

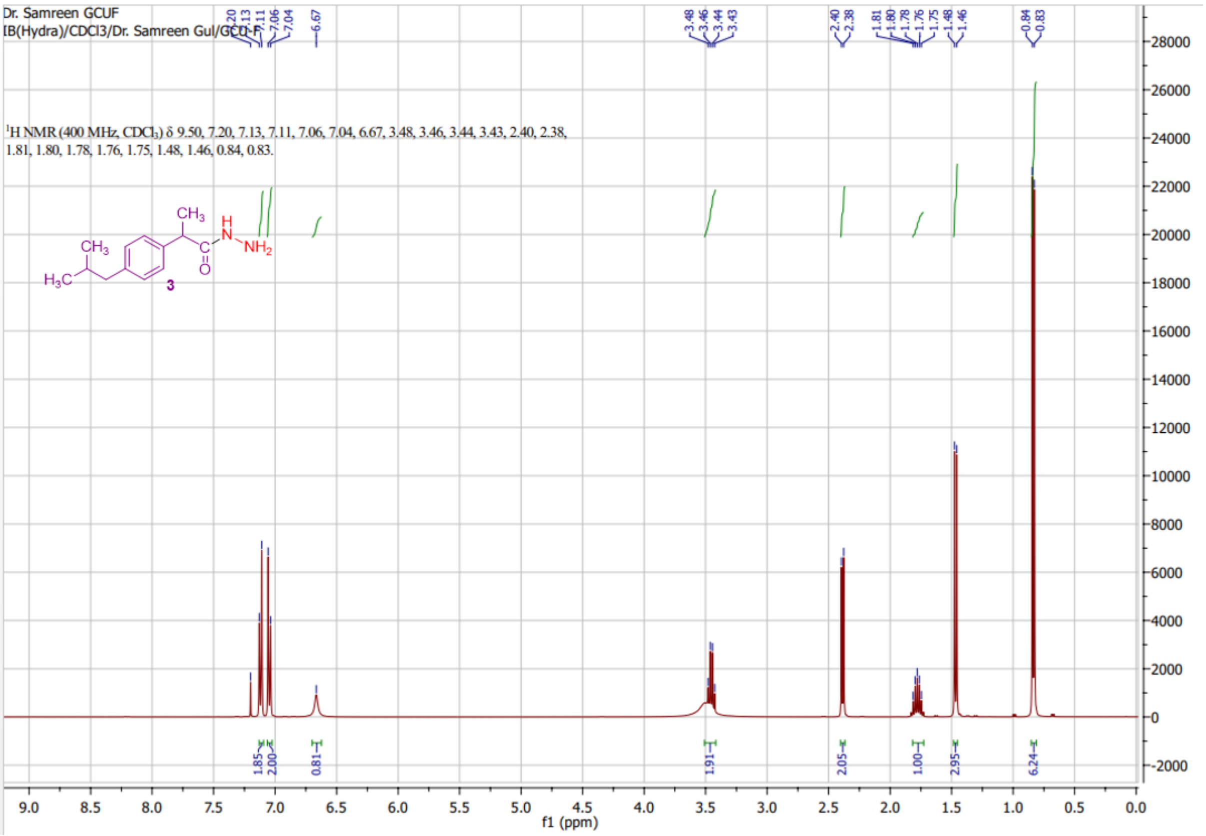

Figure S2: ^{1}H NMR spectrum of compound **3** (Full)

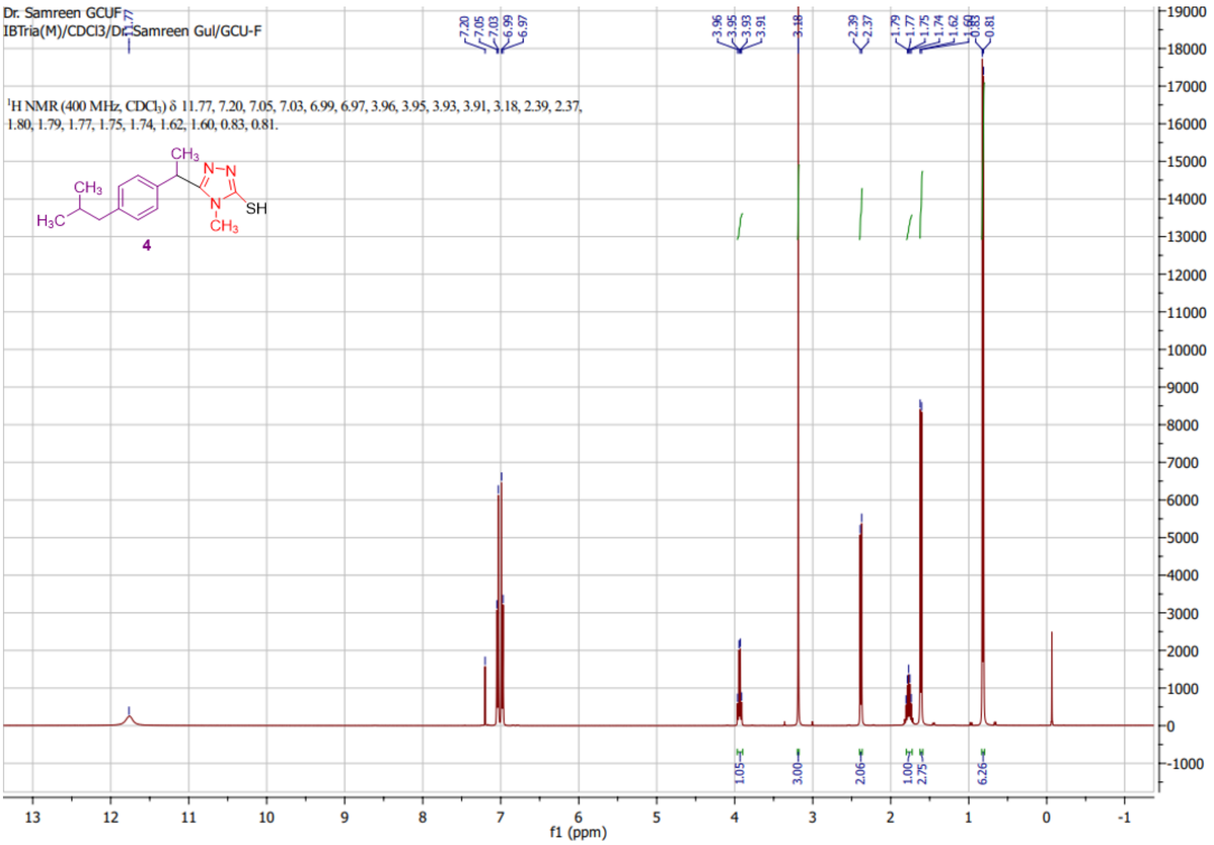

Figure S3: ^{1}H NMR spectrum of compound **4**(Full)

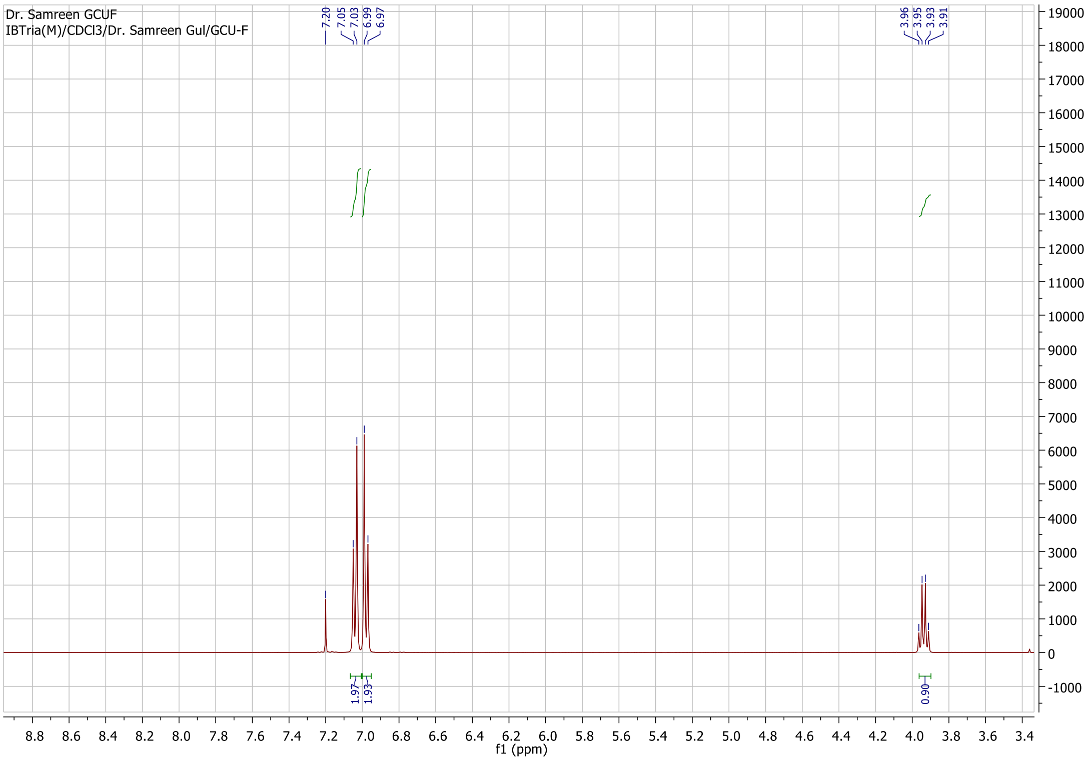

Figure S4. ^{1}H NMR spectrum of compound **4** (aromatic region)

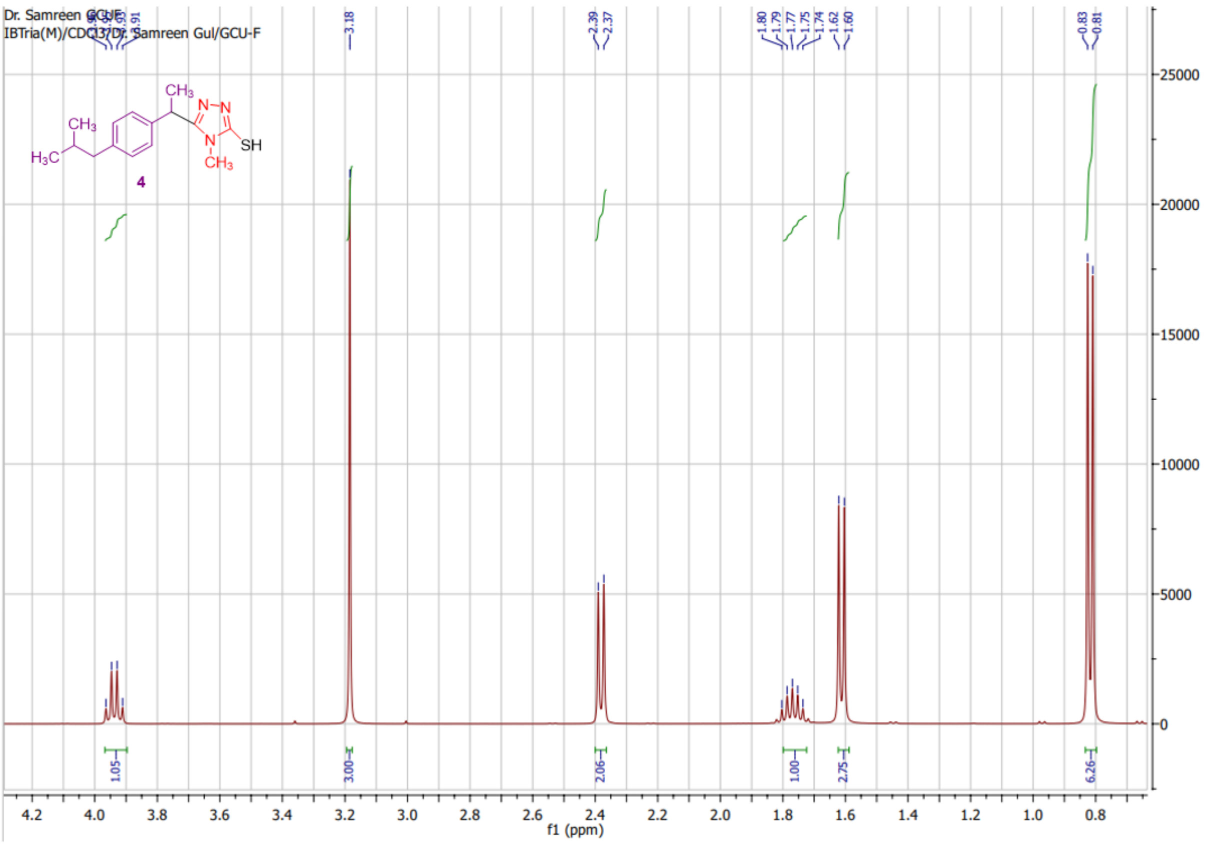

Figure S5. ^{1}H NMR spectrum of compound **4** (aliphatic region)

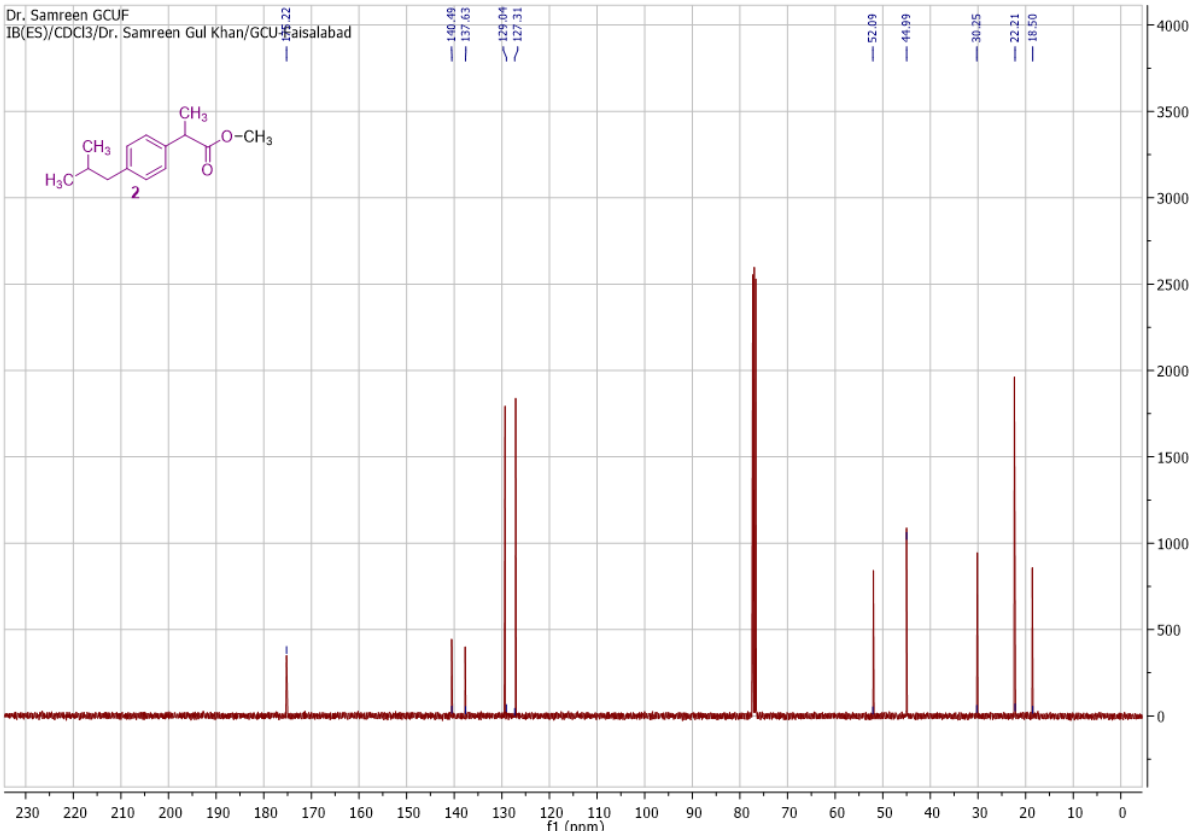

Figure S6. ^{13}C NMR spectrum of compound **2**

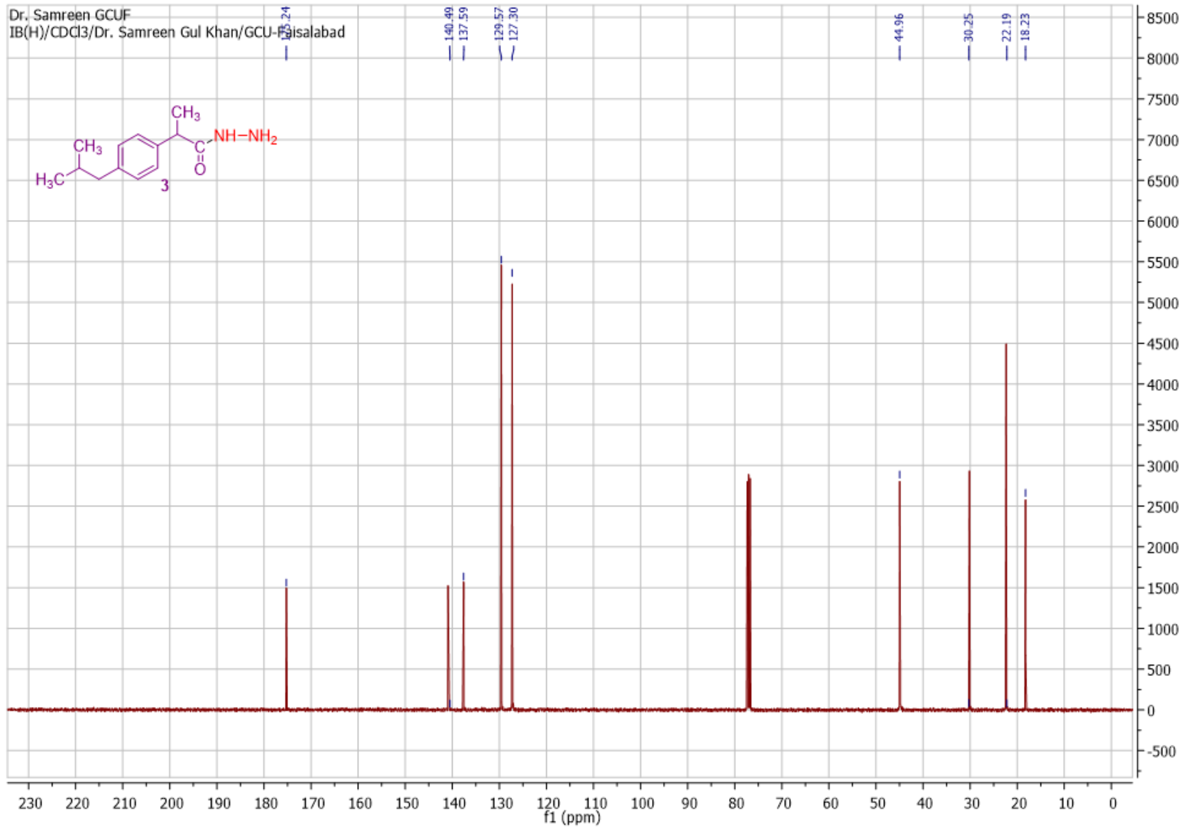

Figure S7. ^{13}C NMR spectrum of compound **3**

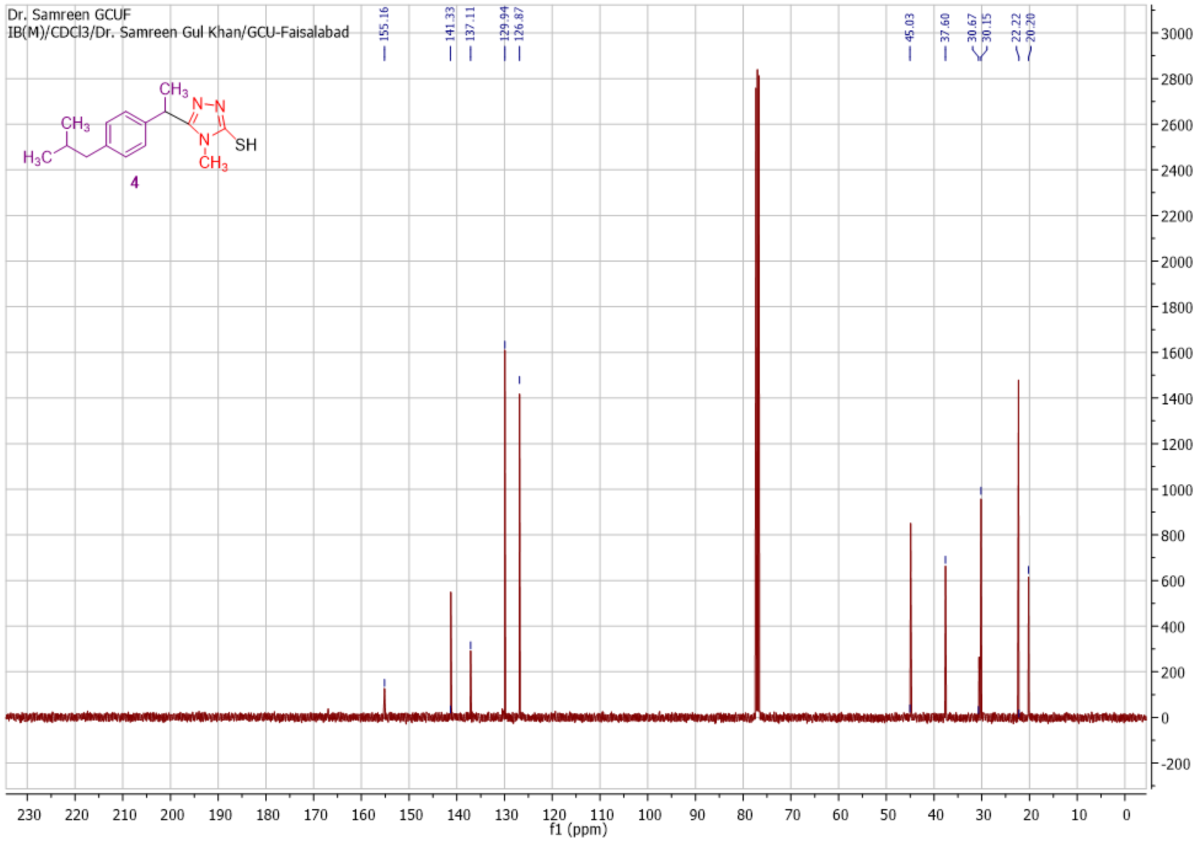

Figure S8. ^{13}C NMR spectrum of compound **4**

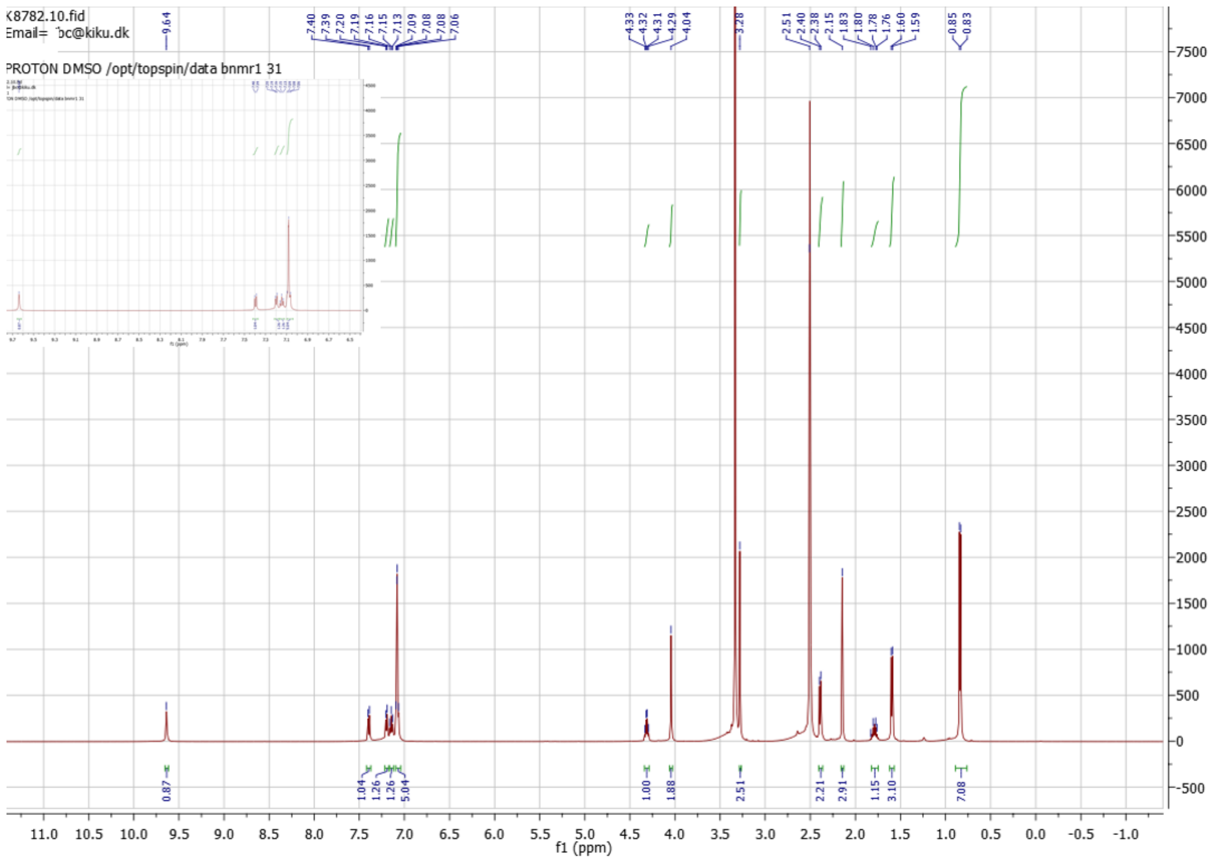

Figure S9: ^{1}H NMR spectrum of compound **7a** (Full)

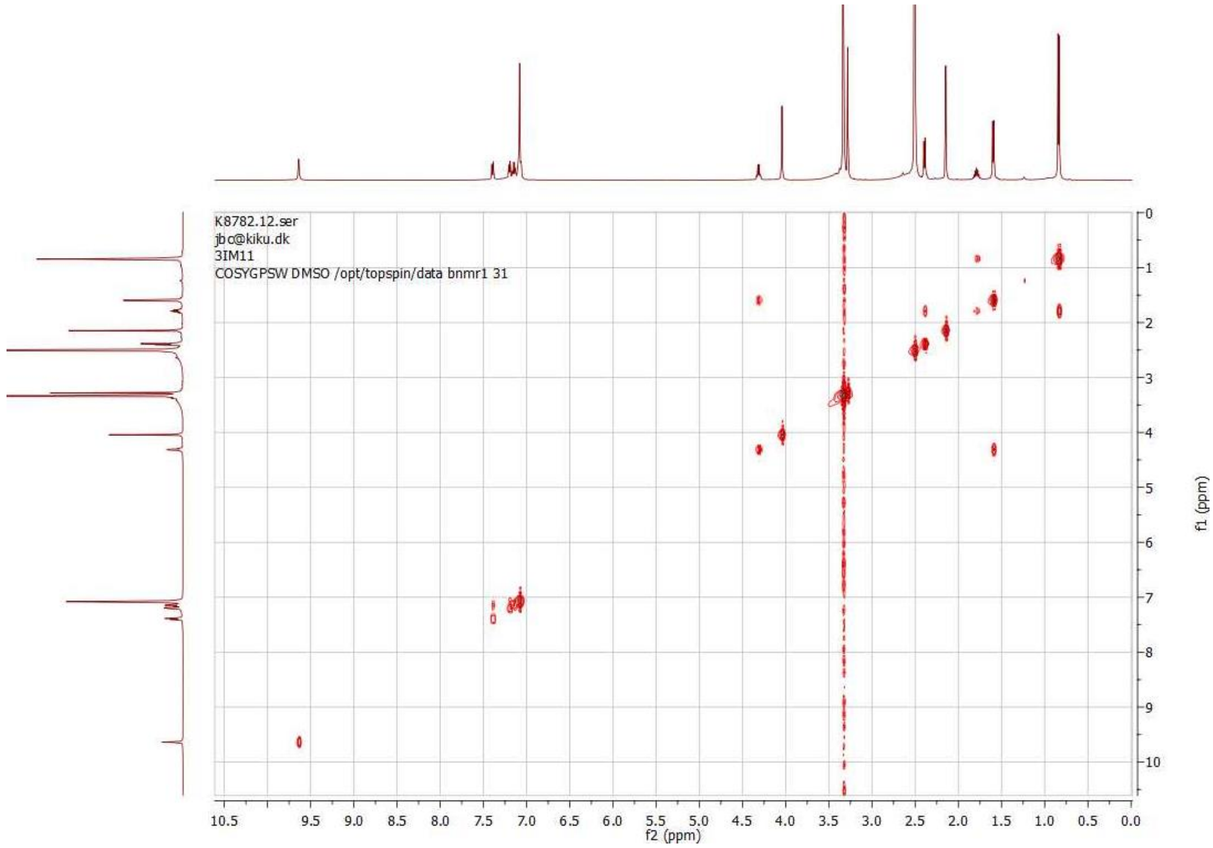

Figure S10. COSY- ^{1}H NMR spectrum of compound **7a**

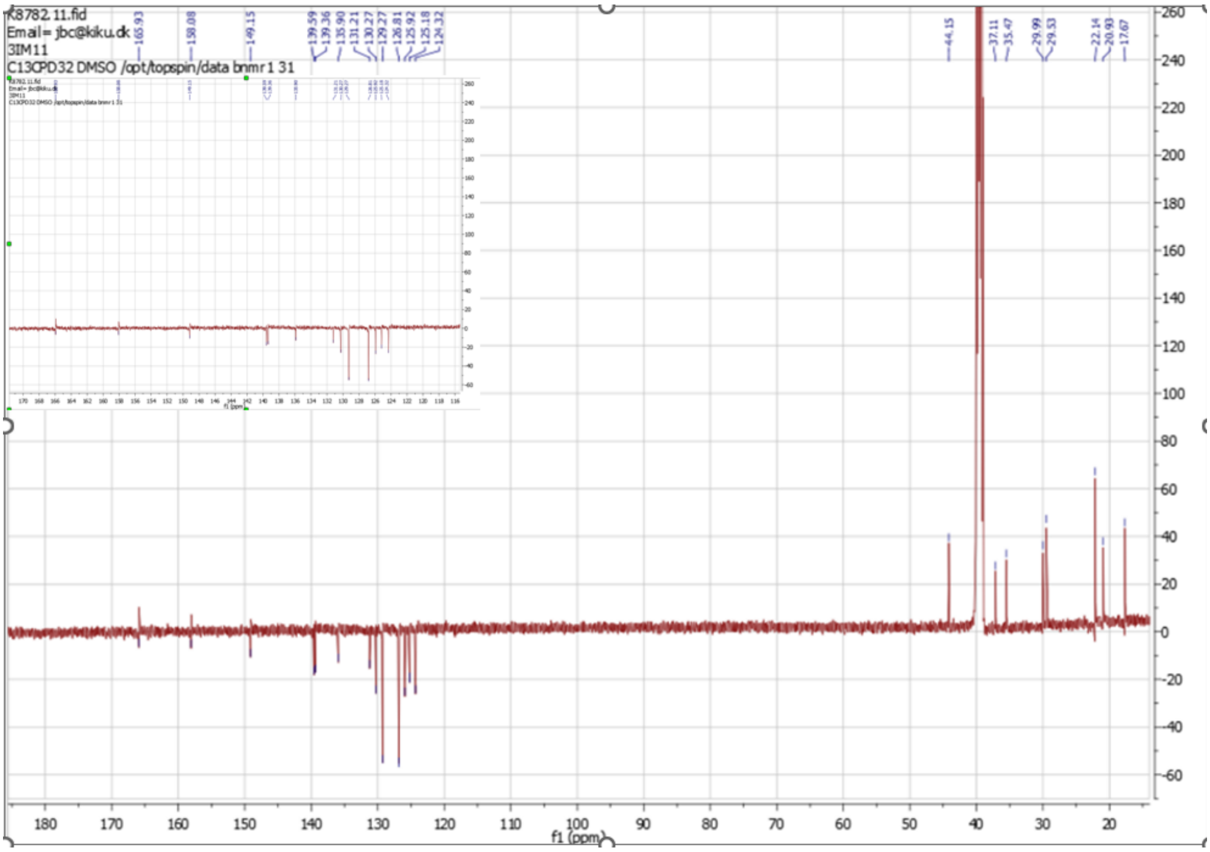

Figure S11. ^{13}C NMR spectrum of compound **7a** (Full spectrum)

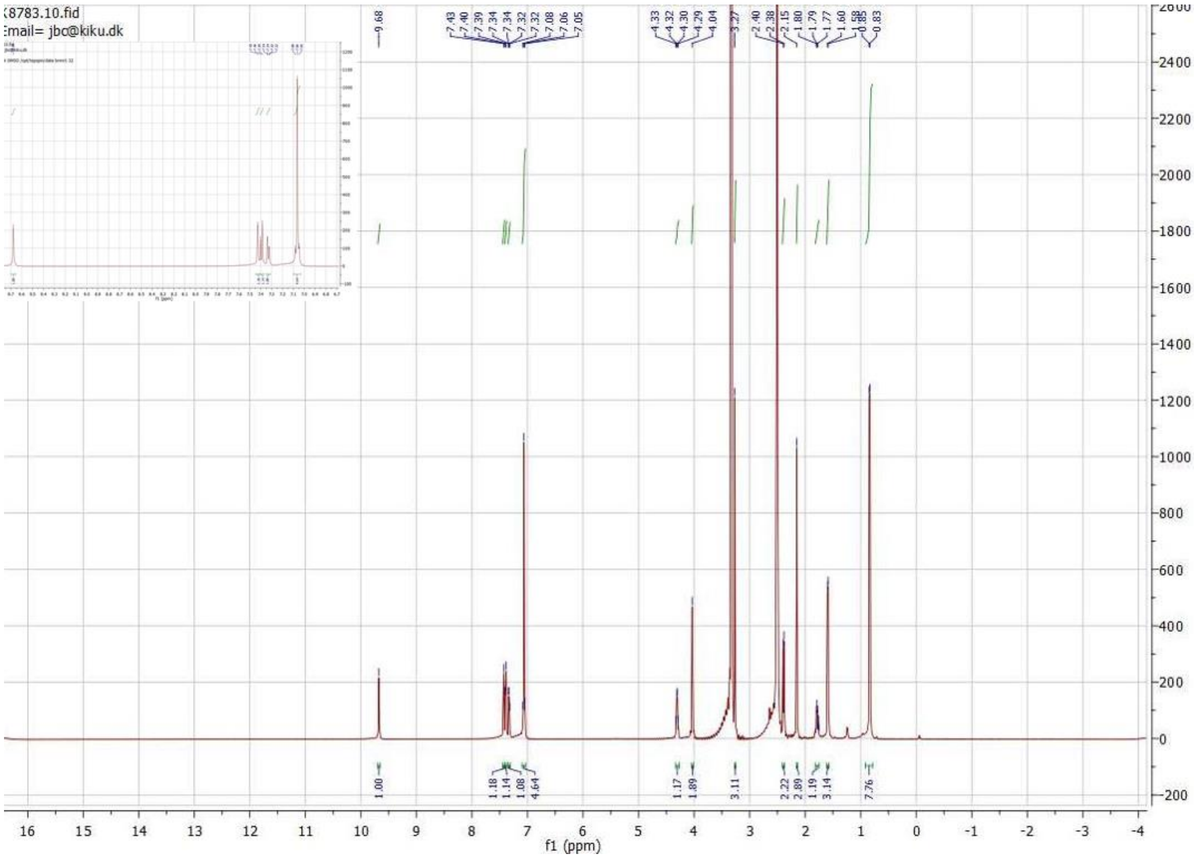

Figure S12: ^{1}H NMR spectrum of compound **7b** (Full)

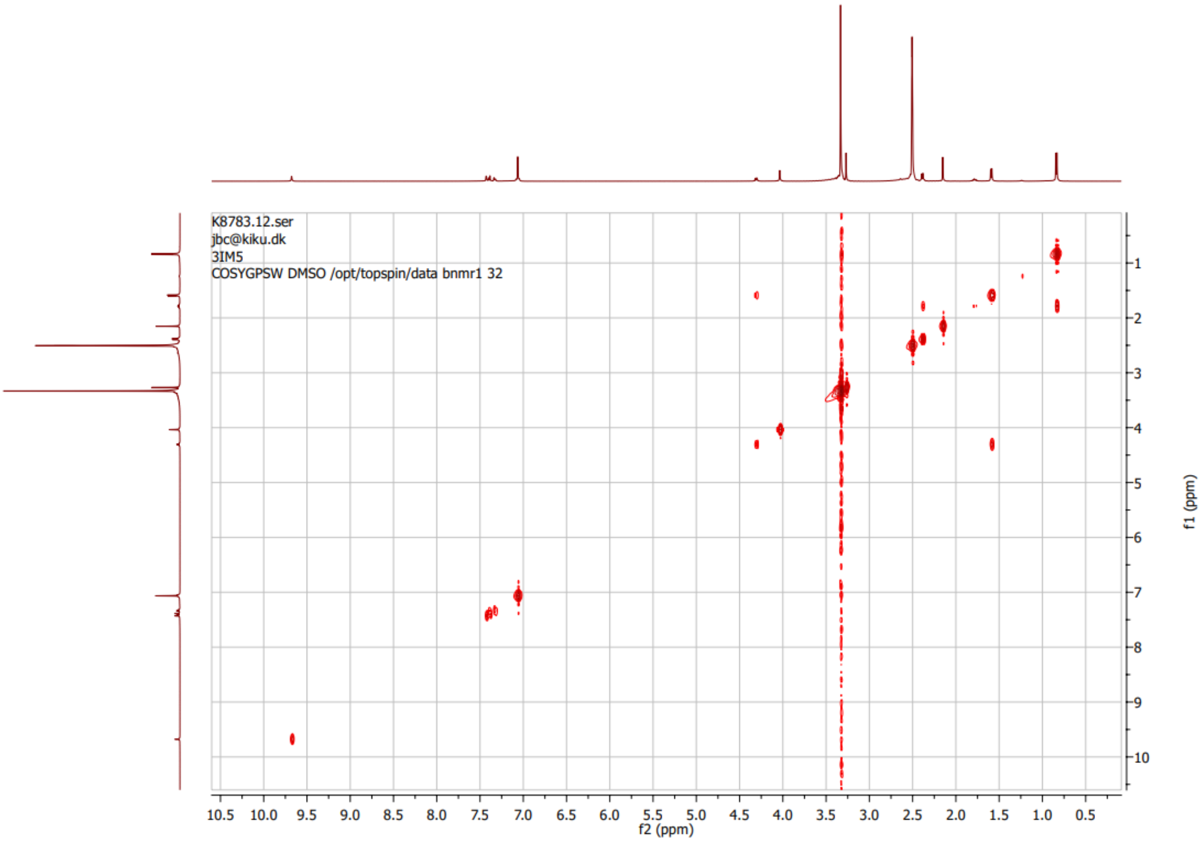

Figure S13. COSY-^{1}H NMR spectrum of compound **7b**

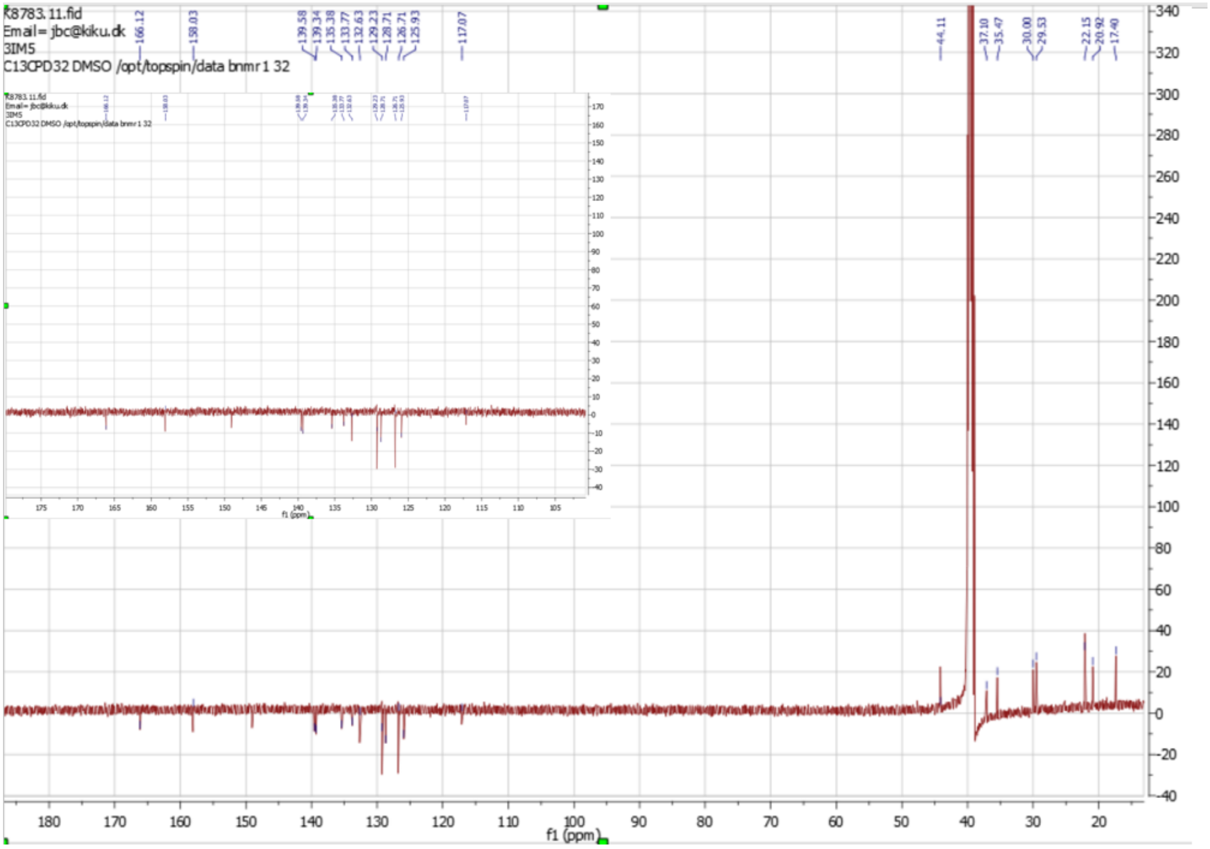

Figure S14. ^{13}C NMR spectrum of compound **7b** (Full spectrum)

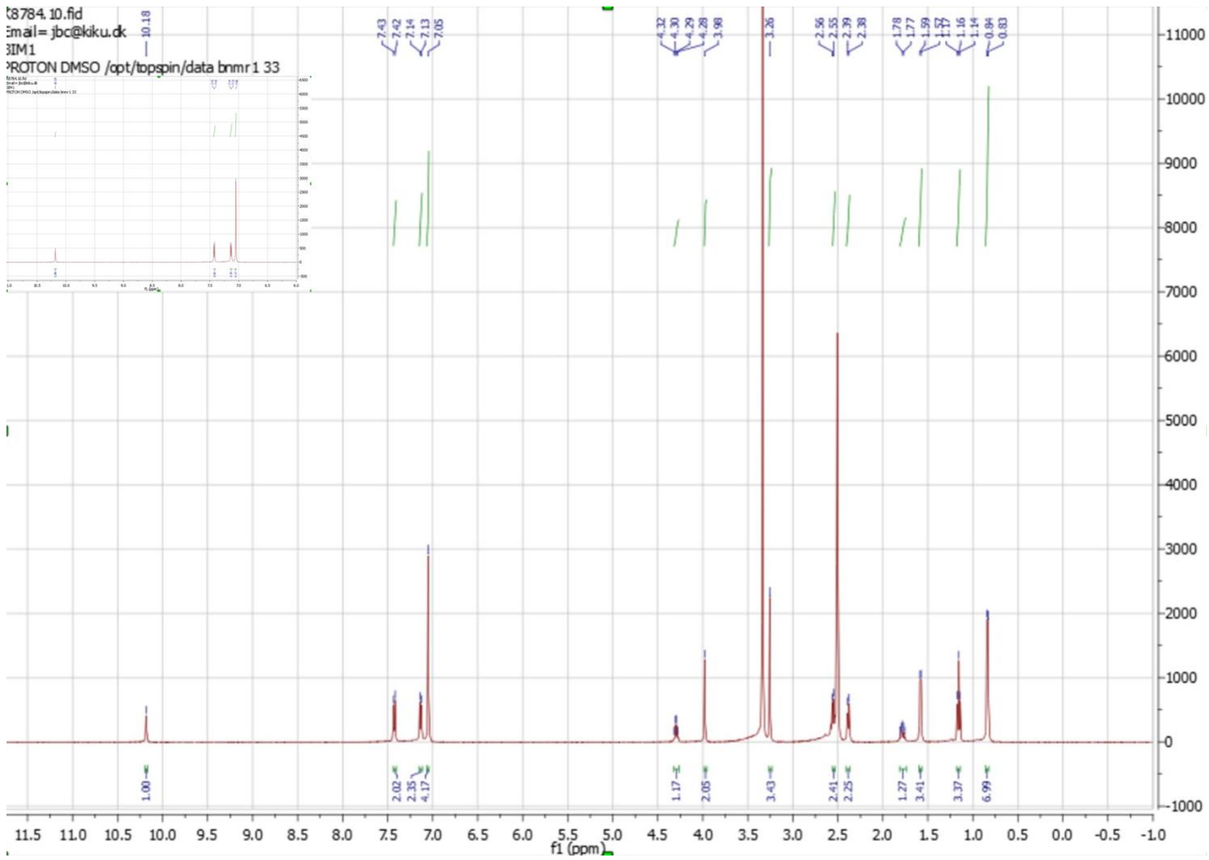

Figure S15. ^{1}H NMR spectrum of compound **7c** (Full spectrum)

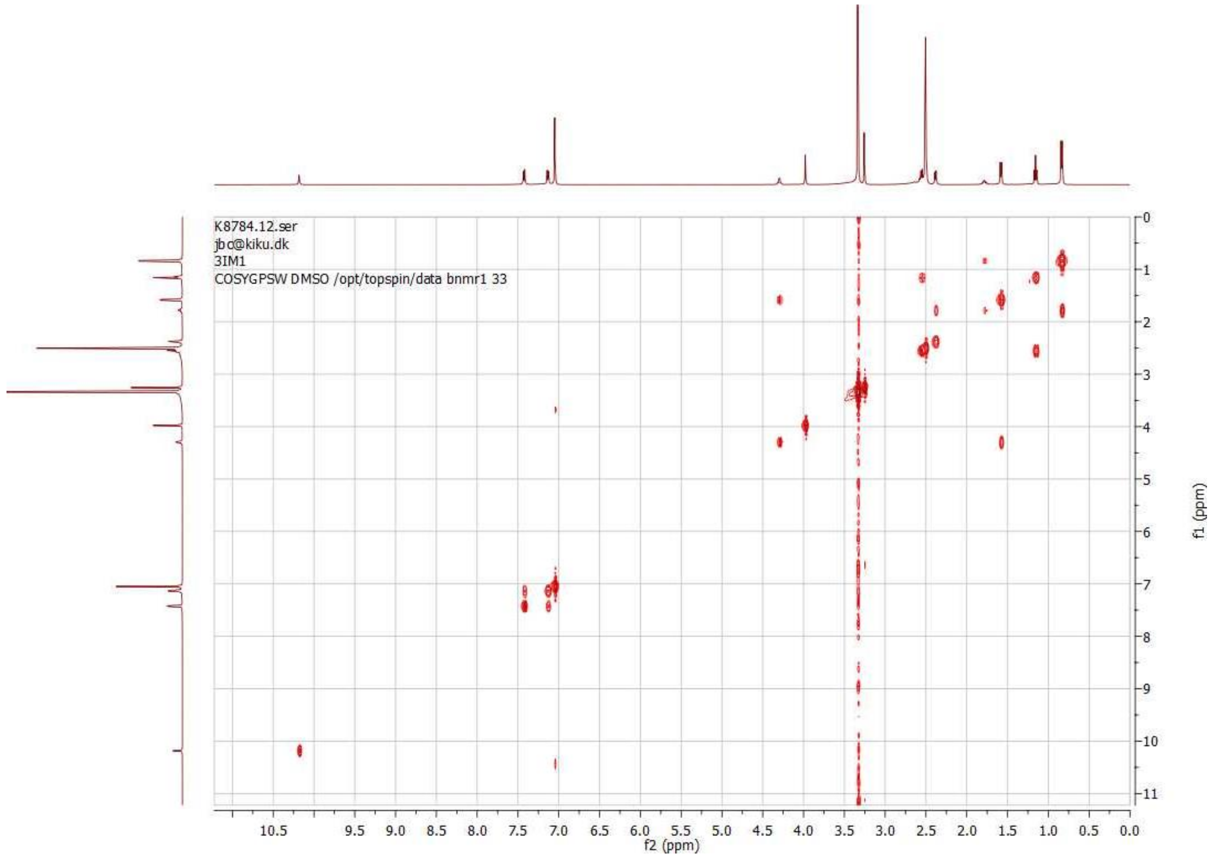

Figure S16. COSY ^{1}H NMR spectrum of compound **7c**

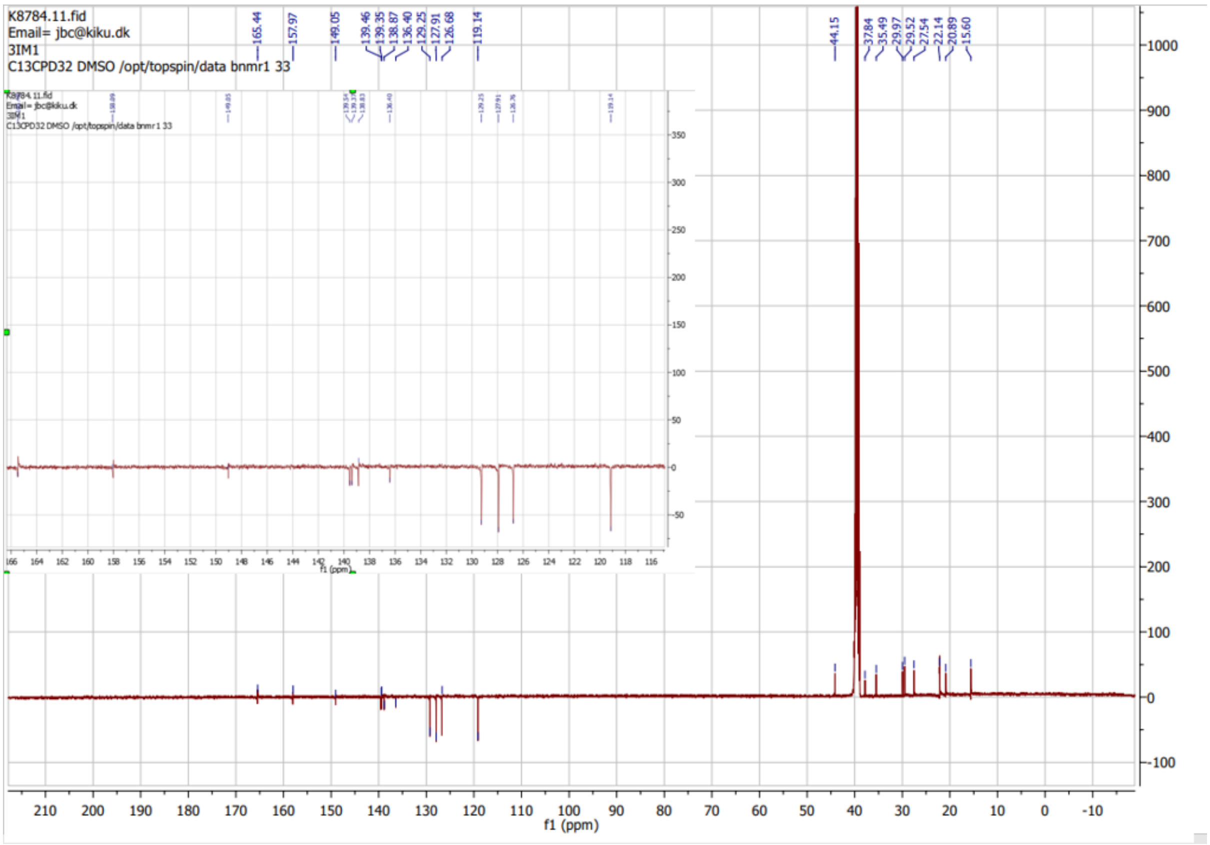

Figure S17. ^{13}C NMR spectrum of compound **7c** (Full spectrum)

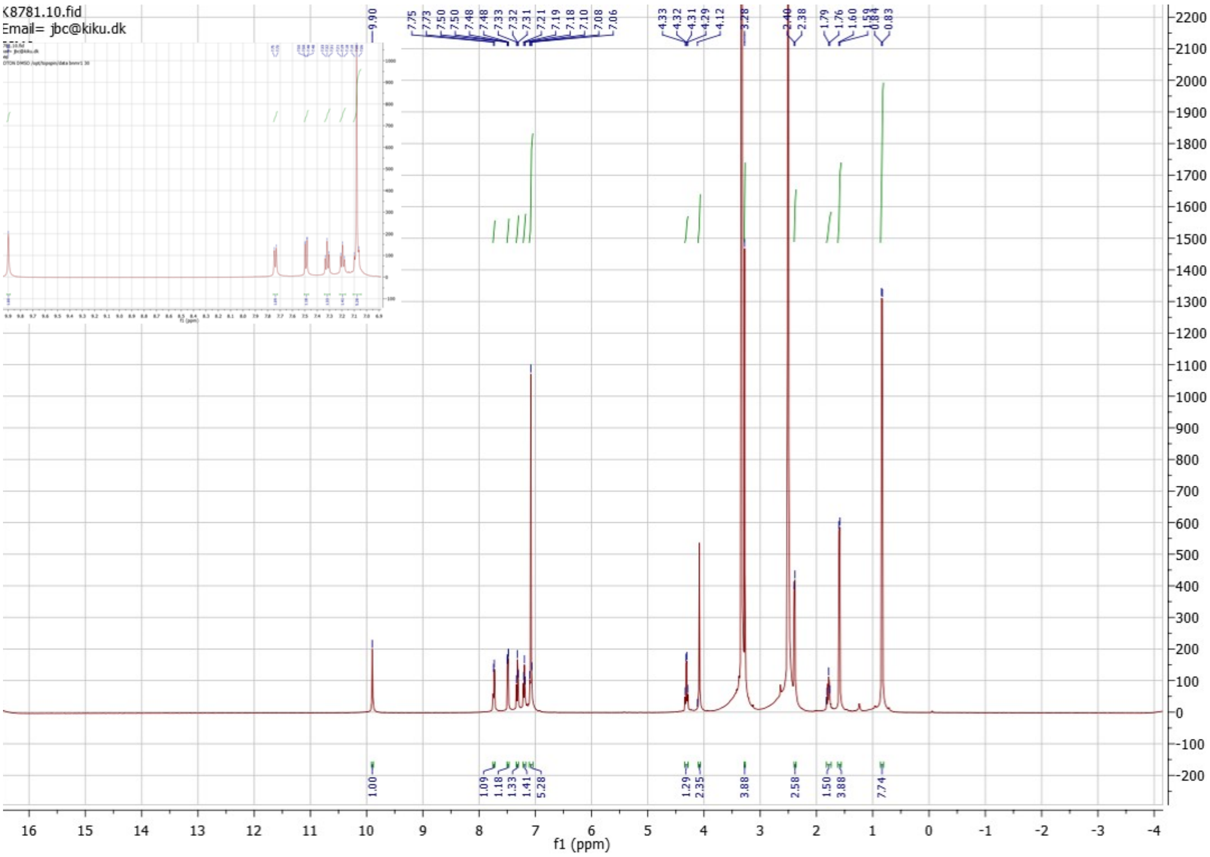

Figure S18: ^{1}H NMR spectrum of compound **7d** (Full

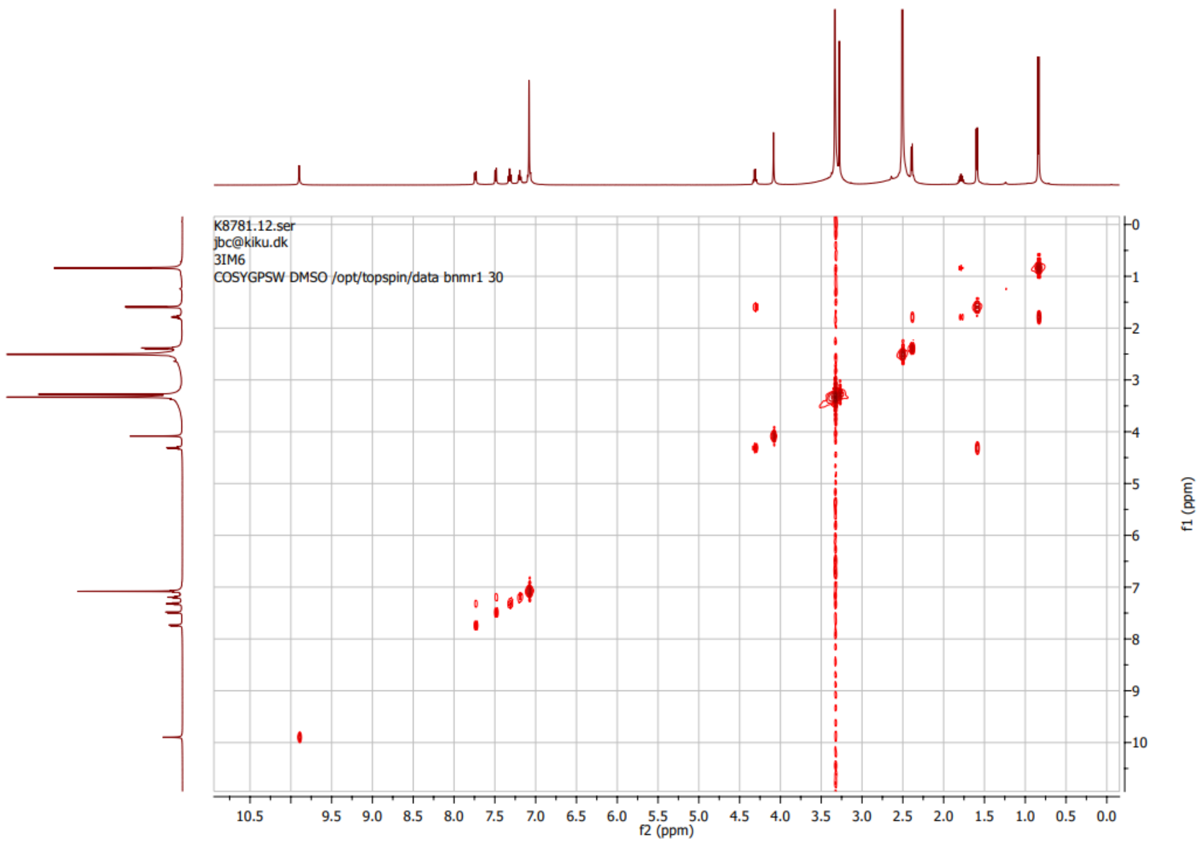

Figure S19. COSY-^{1}H NMR spectrum of compound **7d**

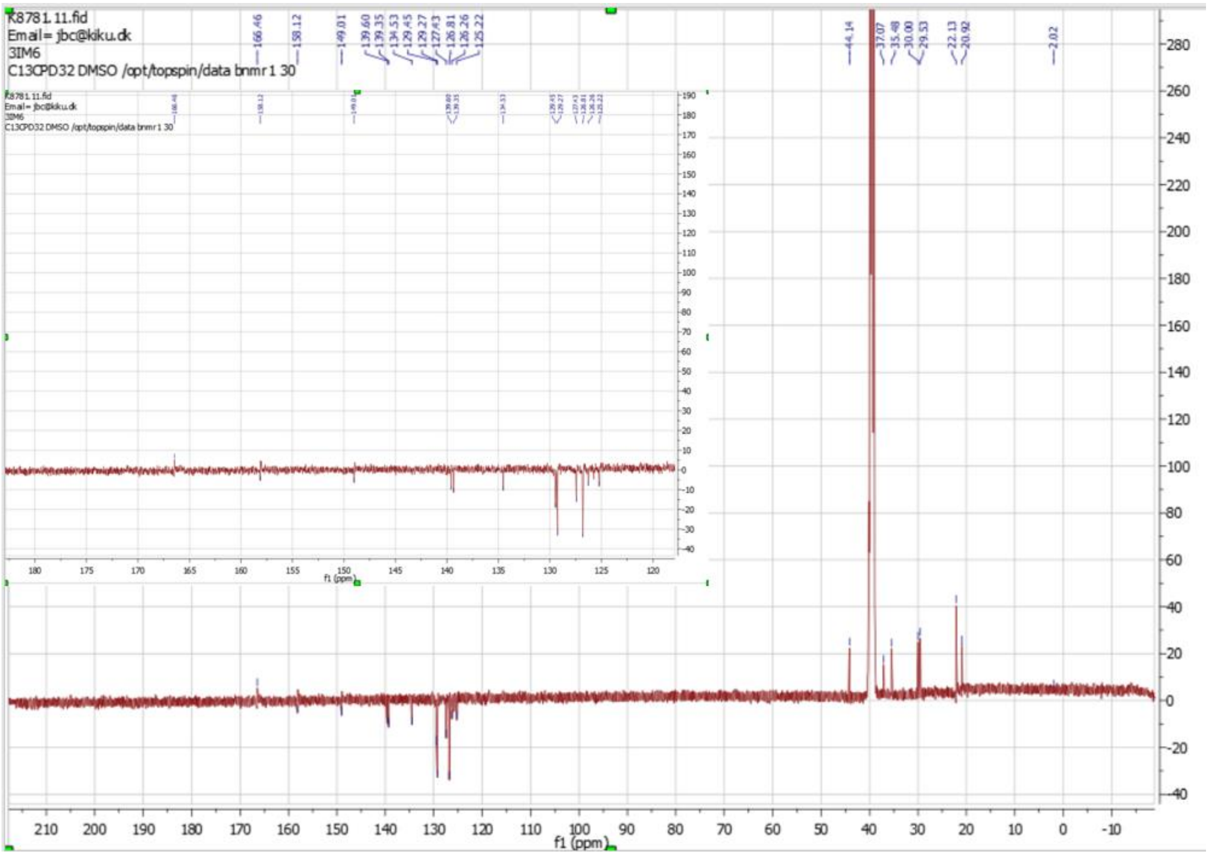

Figure S20. ^{13}C NMR spectrum of compound **7d** (Full spectrum)

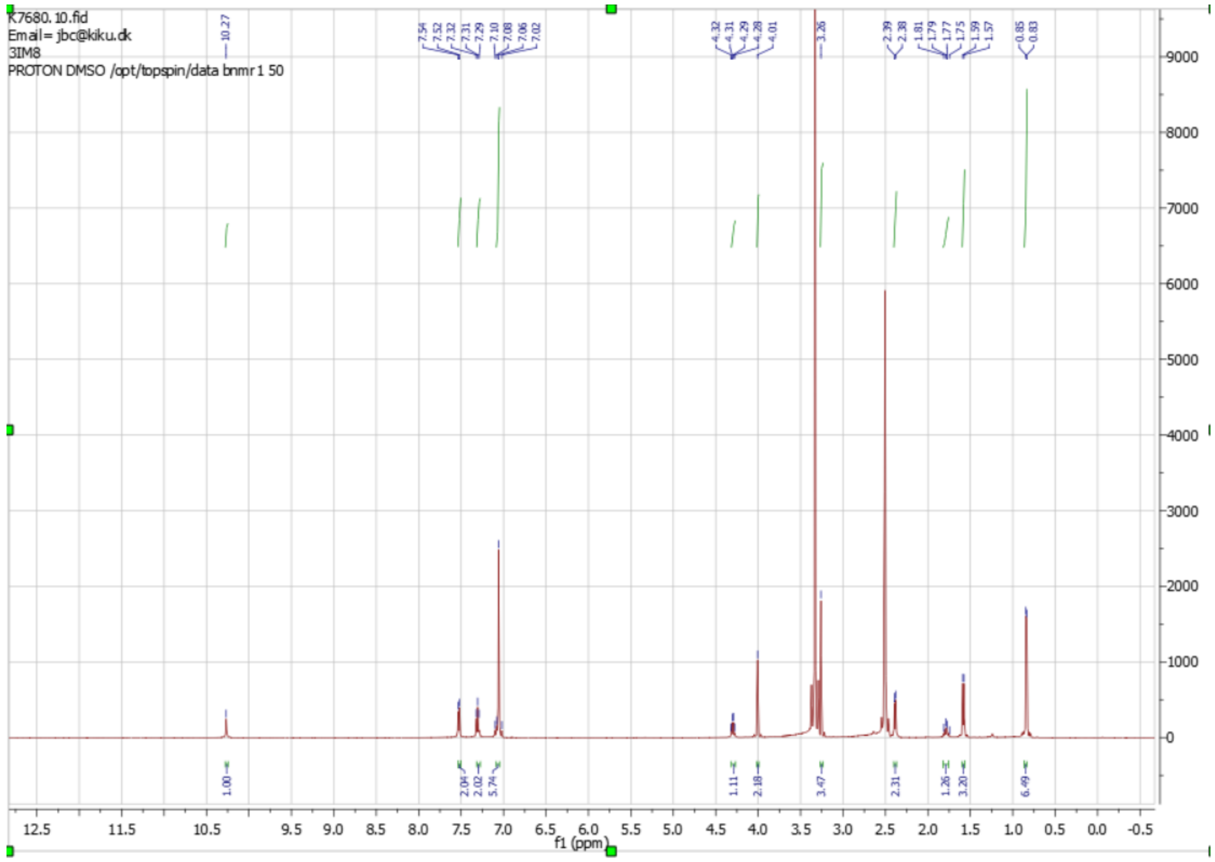

Figure S21: ^{1}H NMR spectrum of compound **7e** (Full)

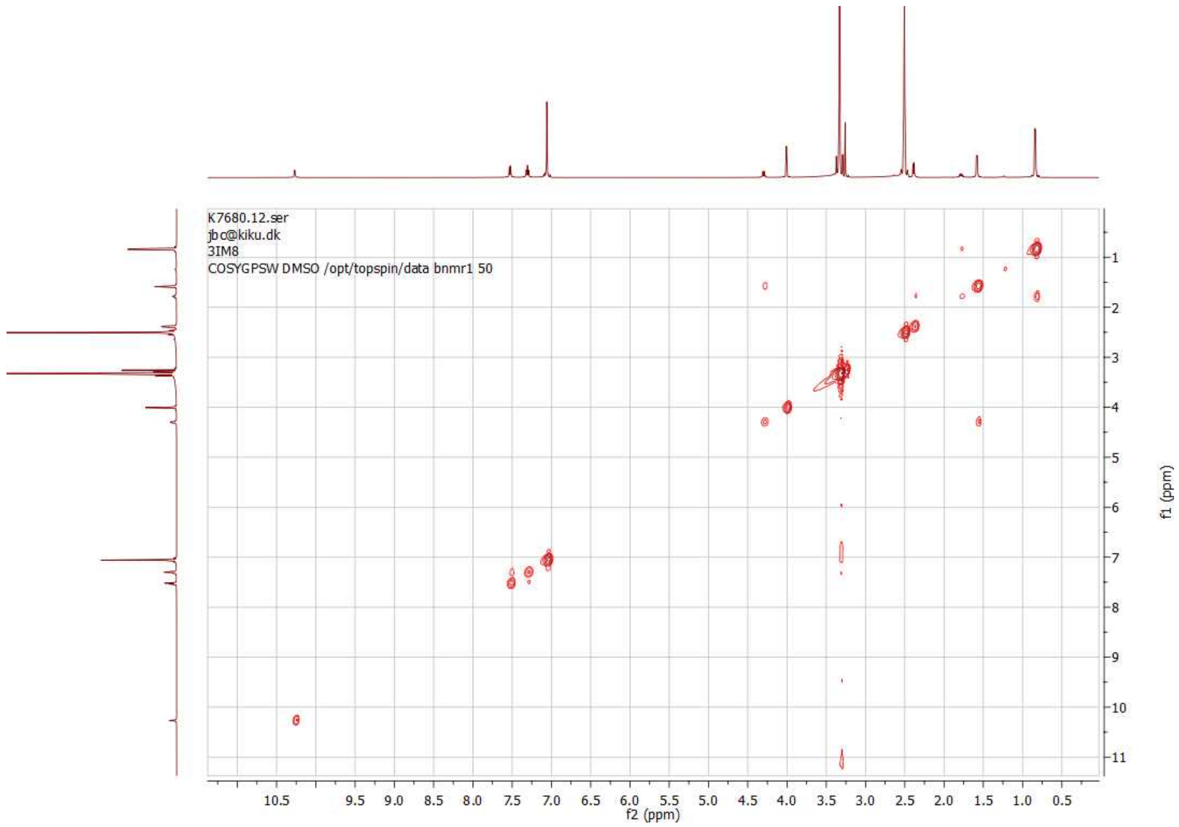

Figure S22. COSY- ^{1}H NMR spectrum of compound **7e**

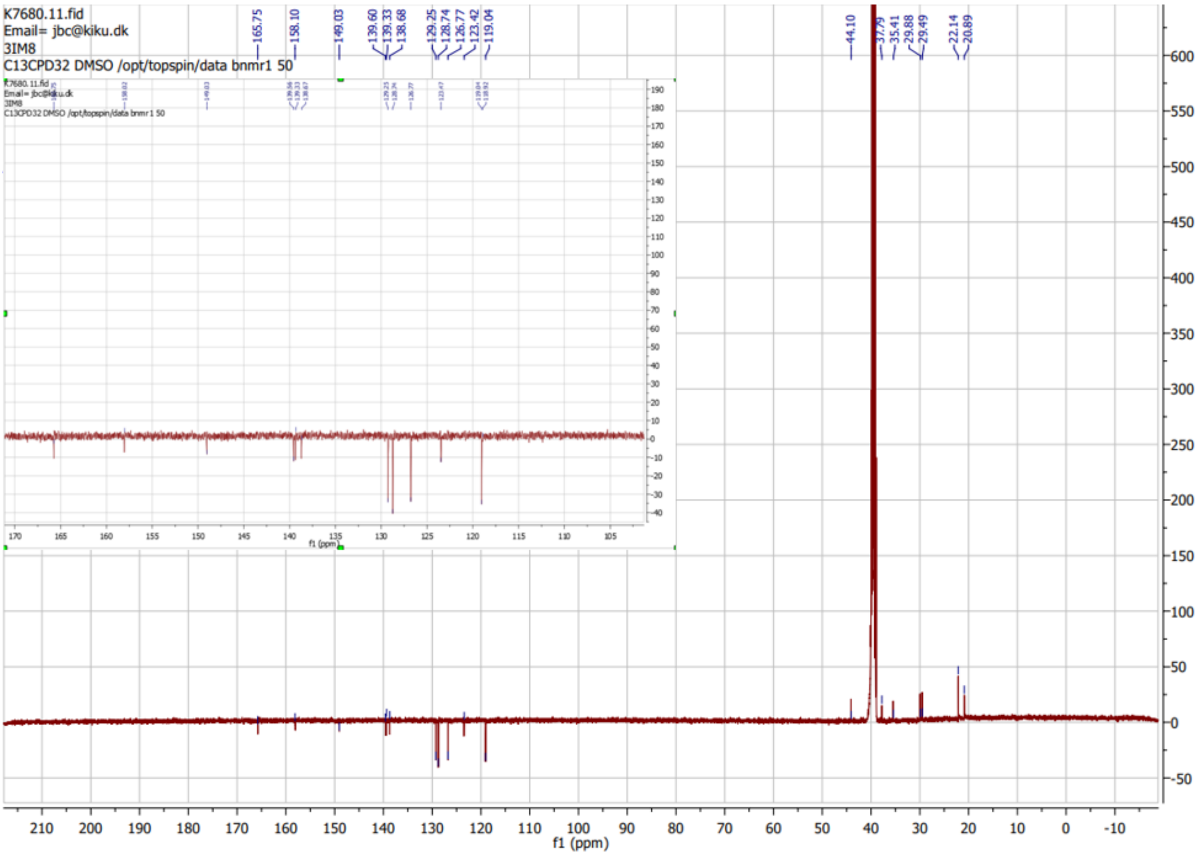

Figure S23. ^{13}C NMR spectrum of compound **7e** (Full spectrum)

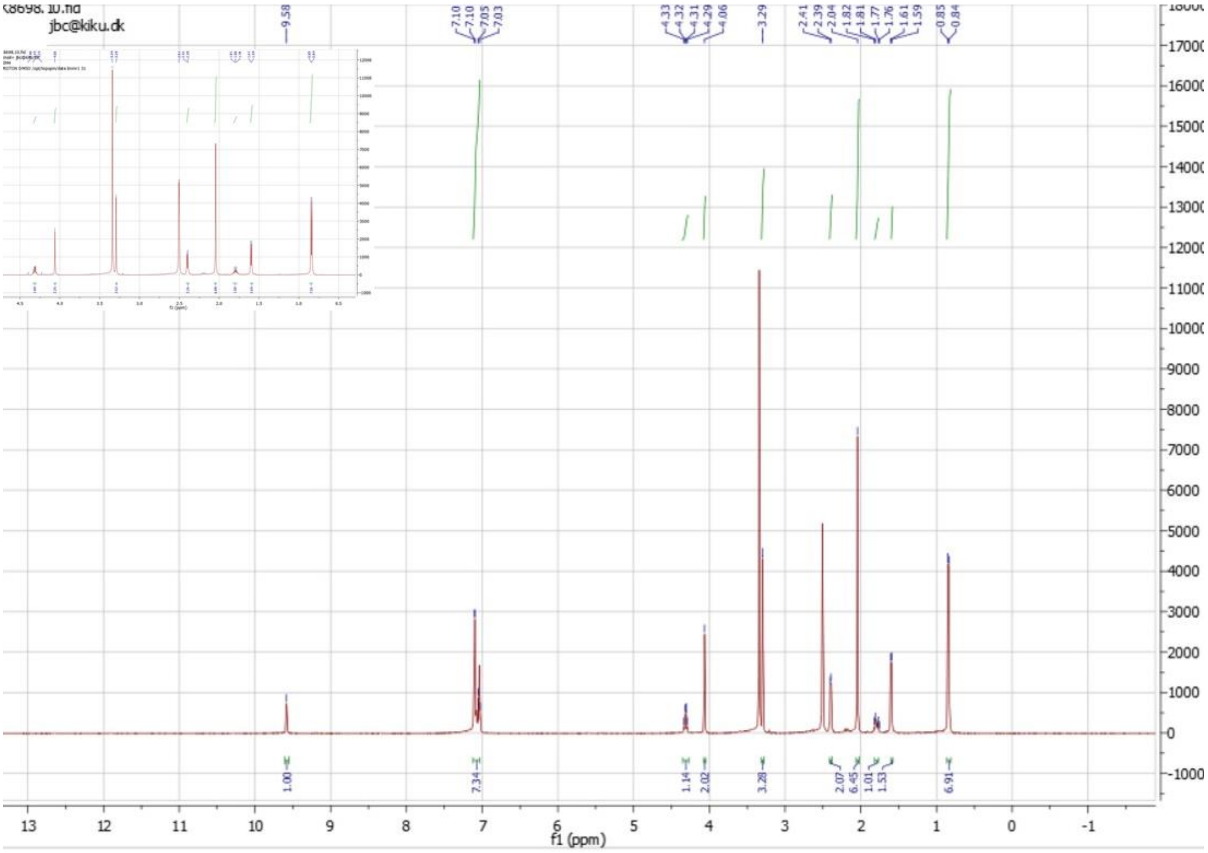

Figure S24: ^{1}H NMR spectrum of compound **7f** (Full)

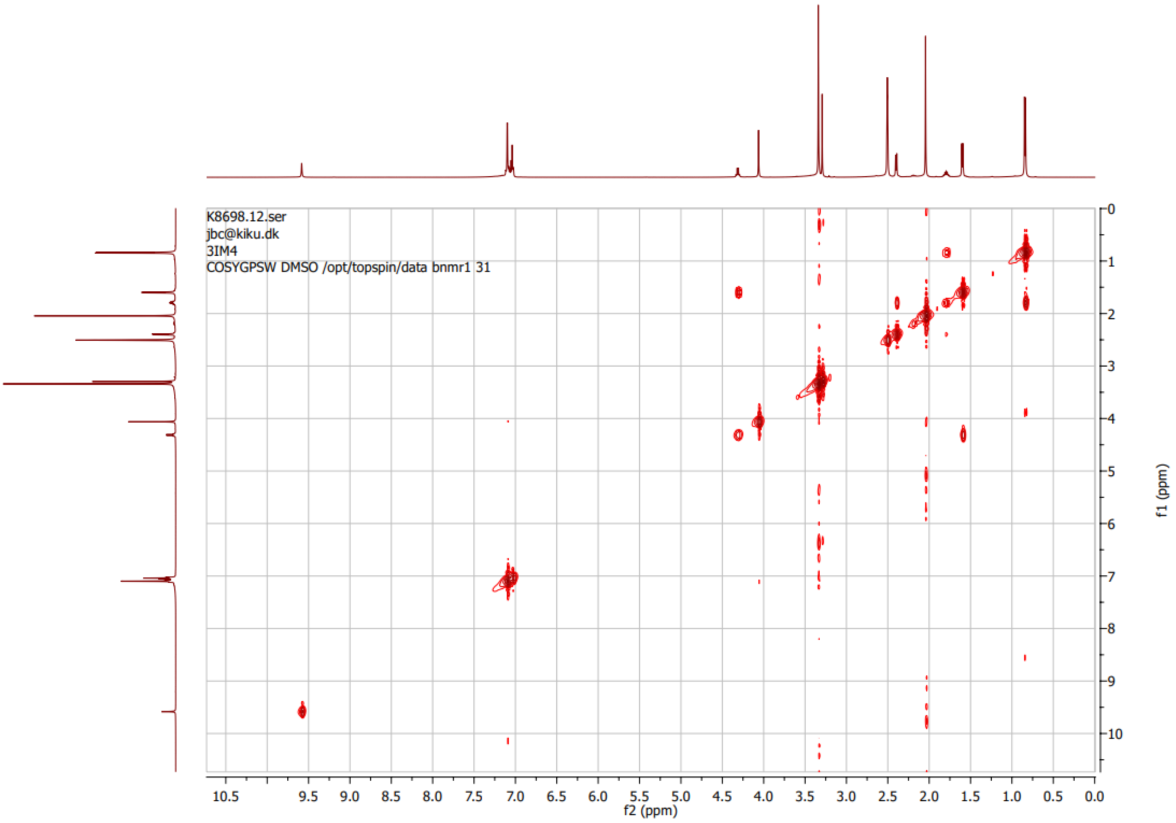

Figure S25. COSY-^{1}H NMR spectrum of compound **7f**

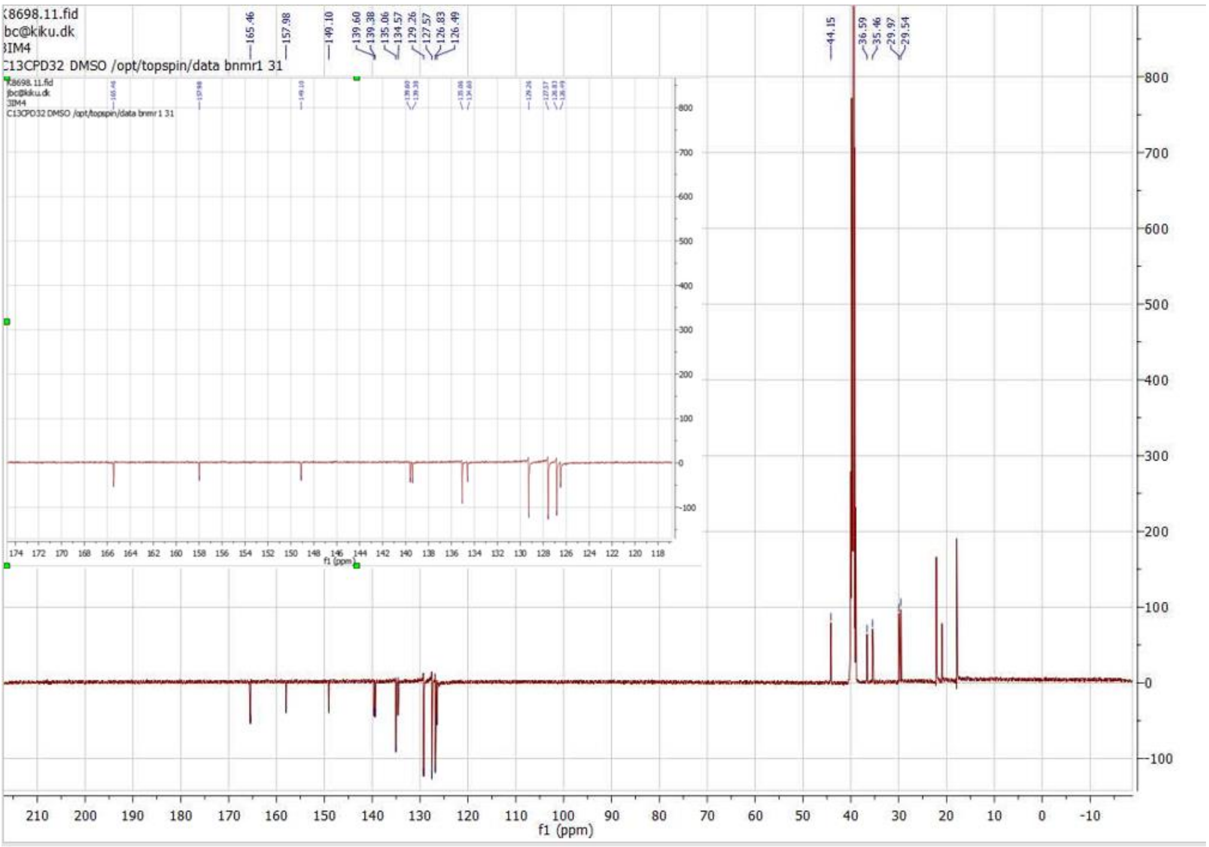

Figure 26. ^{13}C NMR spectrum of compound **7f** (Full spectrum)

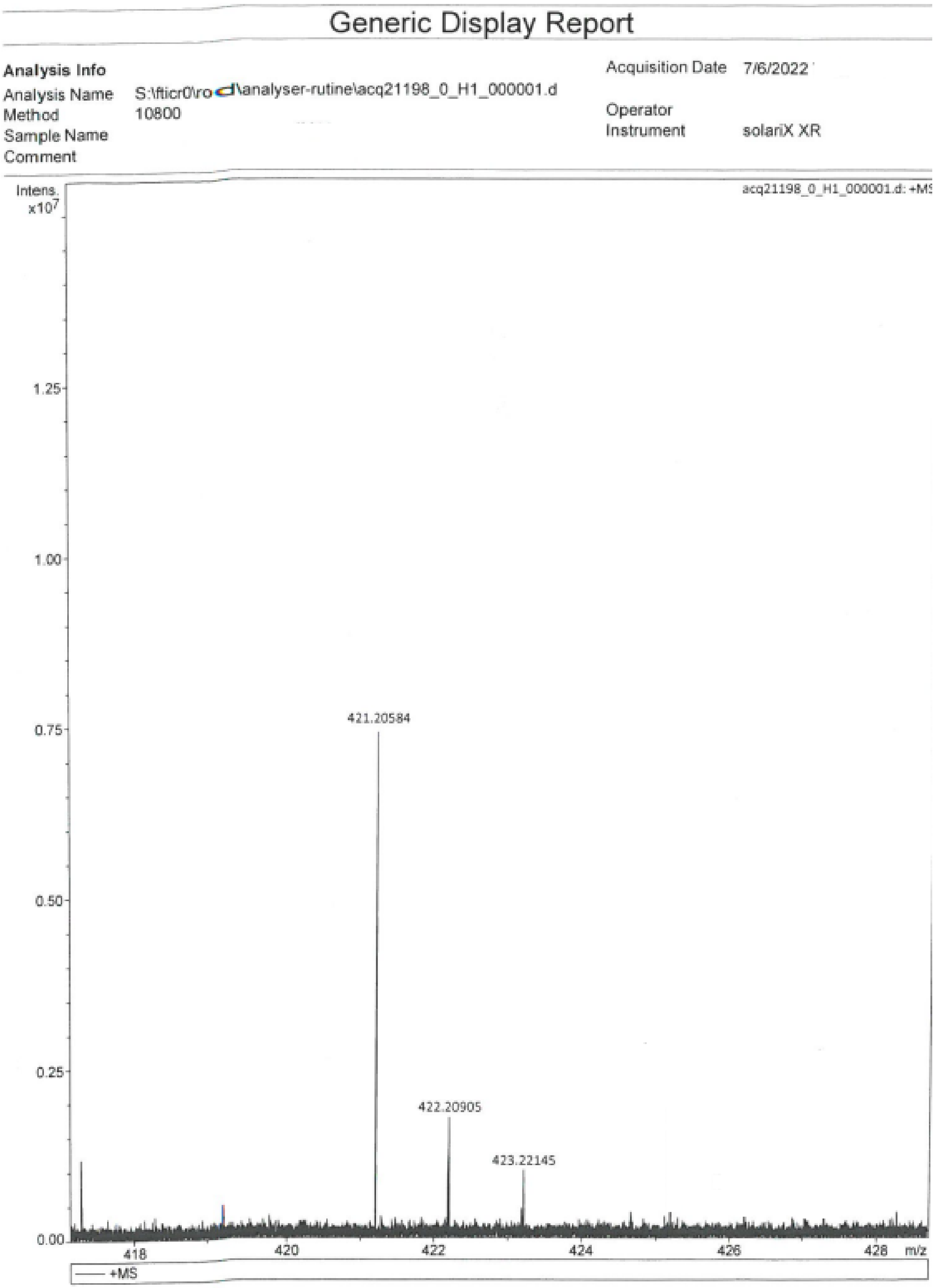

Figure S27. HRMS spectrum of compound **7a**

Generic Display Report

Analysis Info		Acquisition Date	7/6/2022 7:13:14 AM
Analysis Name	S:\fticr0\rod\analyser-rutine\acq21200_0_J1_000001.d		
Method	10800	Operator	
Sample Name		Instrument	solariX XR
Comment			

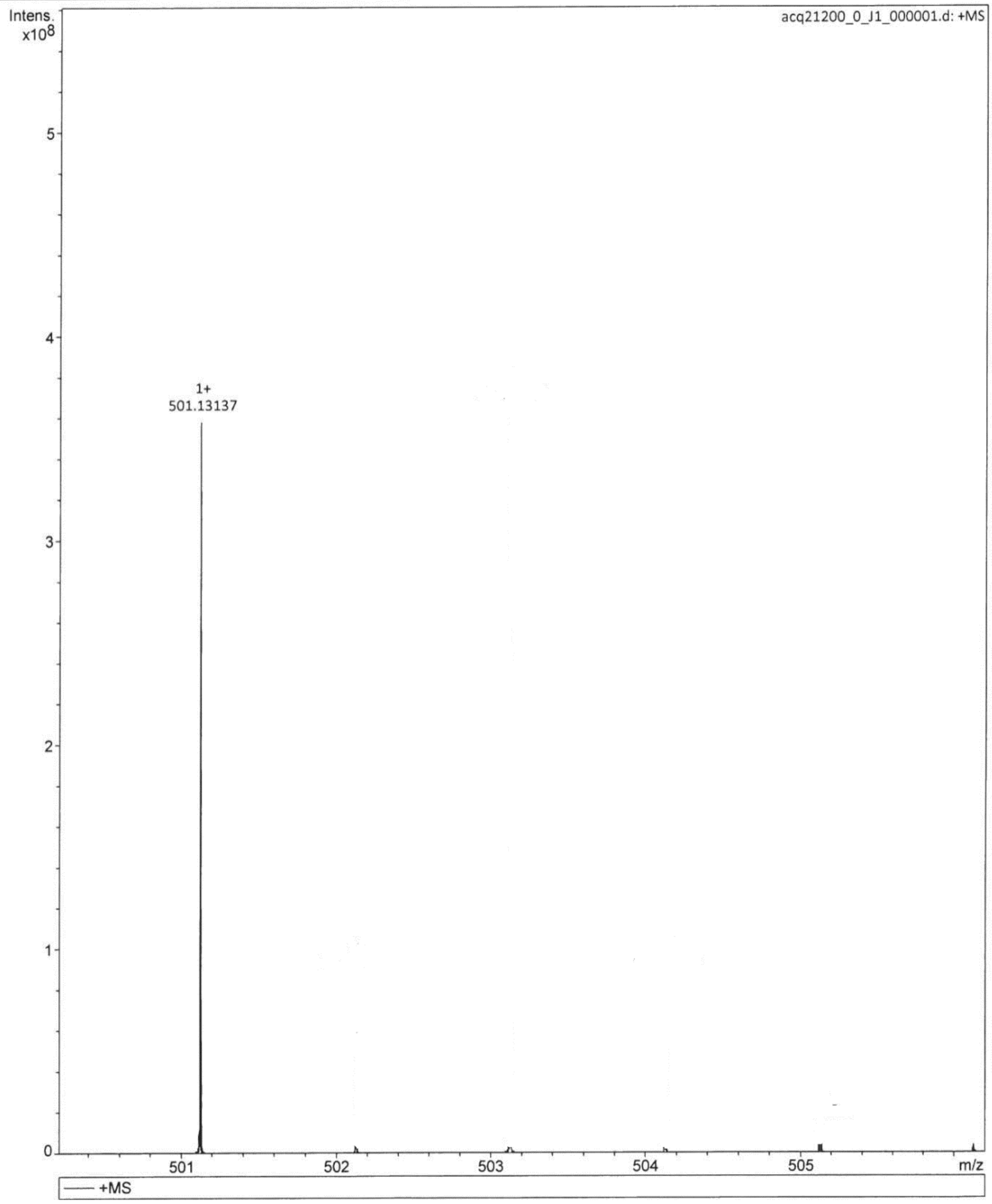

Figure S28. HRMS spectrum of compound **7b**

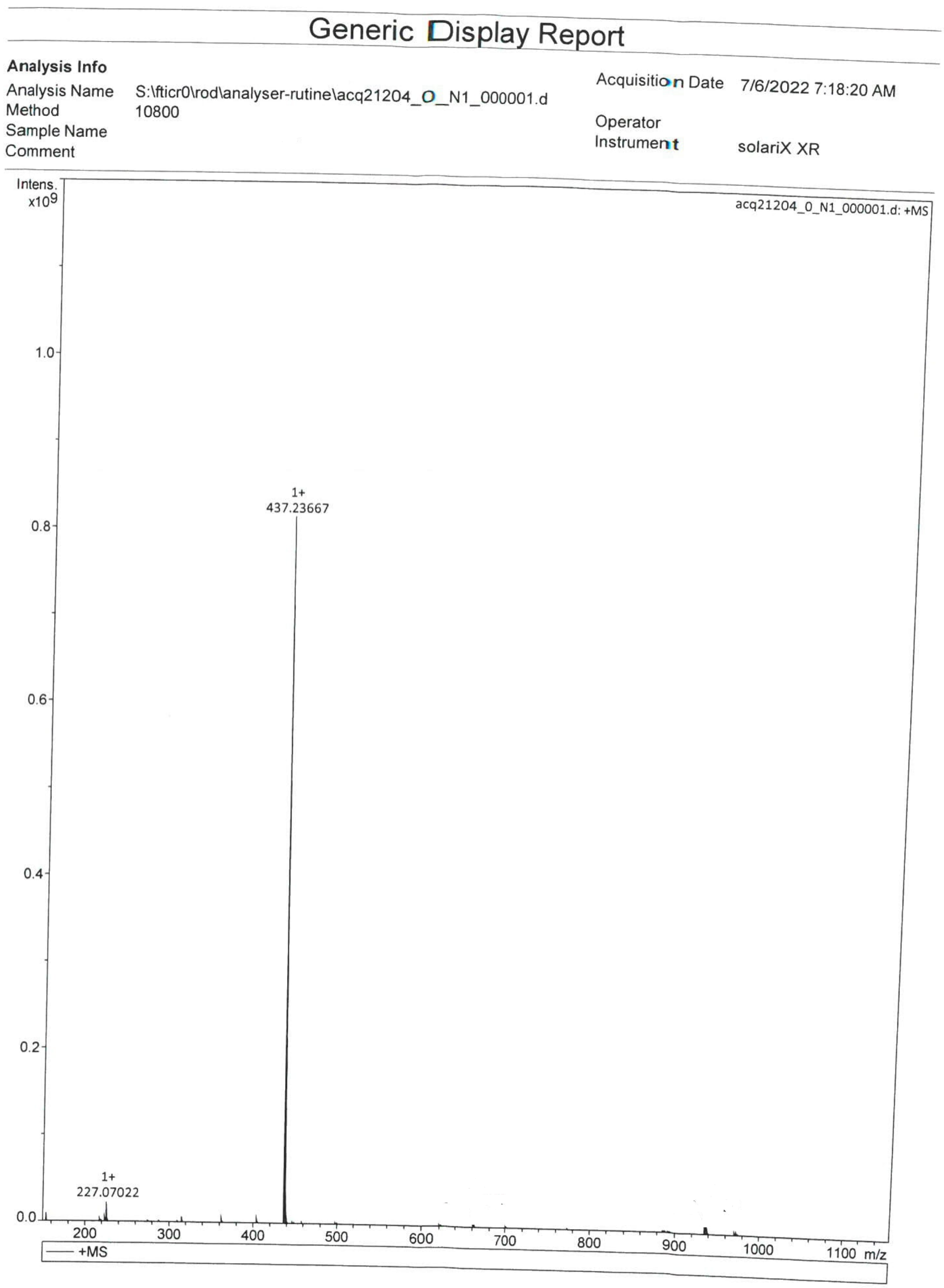

Figure S29. HRMS spectrum of compound **7c**

Generic Display Report

Analysis Info

Analysis Name	S:\fticr0\rod\analyser-rutine\acq21199_0_I1_000001.d	Acquisition Date	7/6/2022 7:12:06 AM
Method	10800	Operator	
Sample Name		Instrument	solariX XR
Comment			

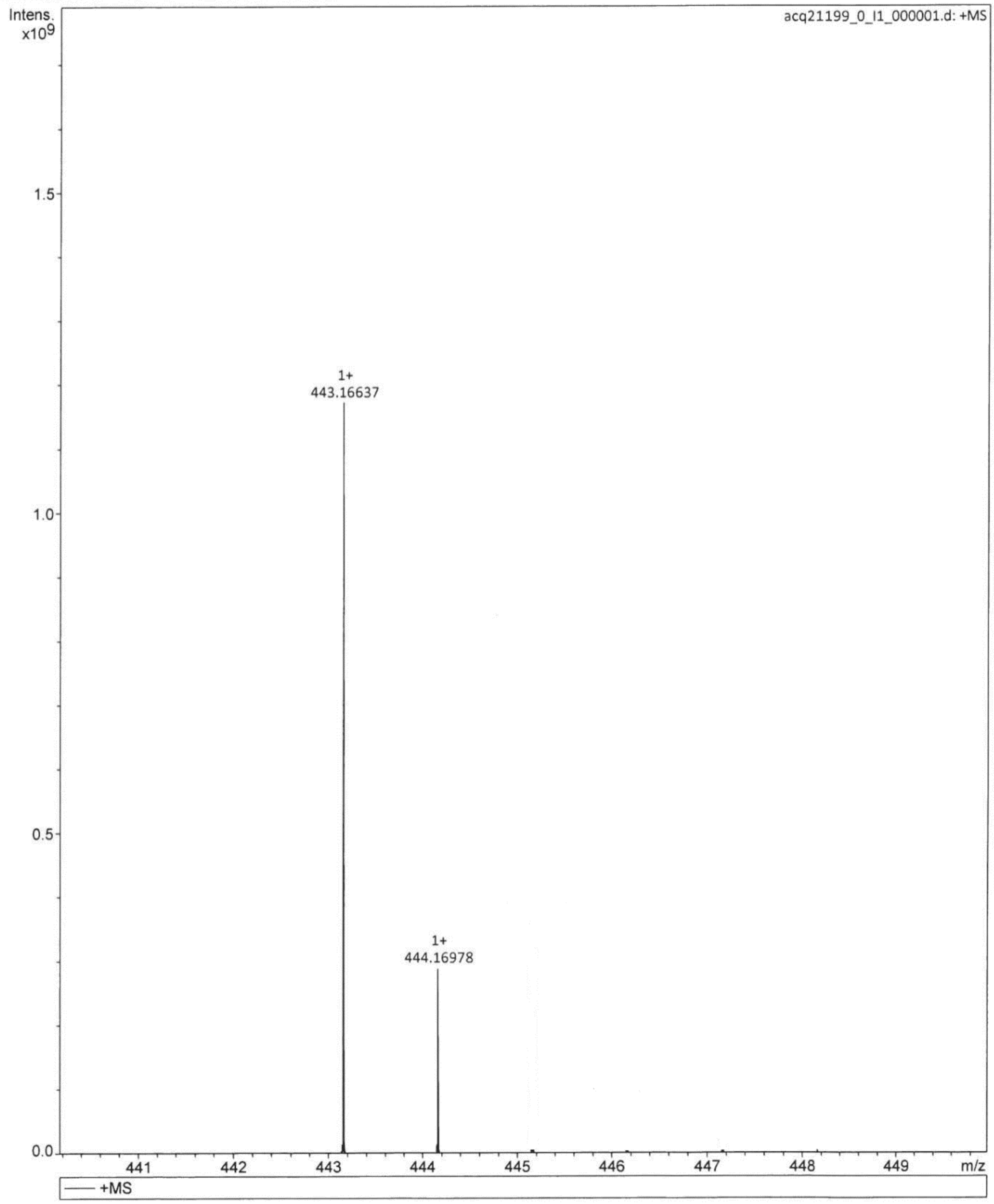

Figure S30. HRMS spectrum of compound **7d**

Generic Display Report

Analysis Info

Analysis Name	S:\fticr0\rod\analyser-rutine\acq21203_0_M1_000001.d
Method	10800
Sample Name	
Comment	
Acquisition Date	7/6/2022 7:16:58 AM
Operator	
Instrument	solariX XR

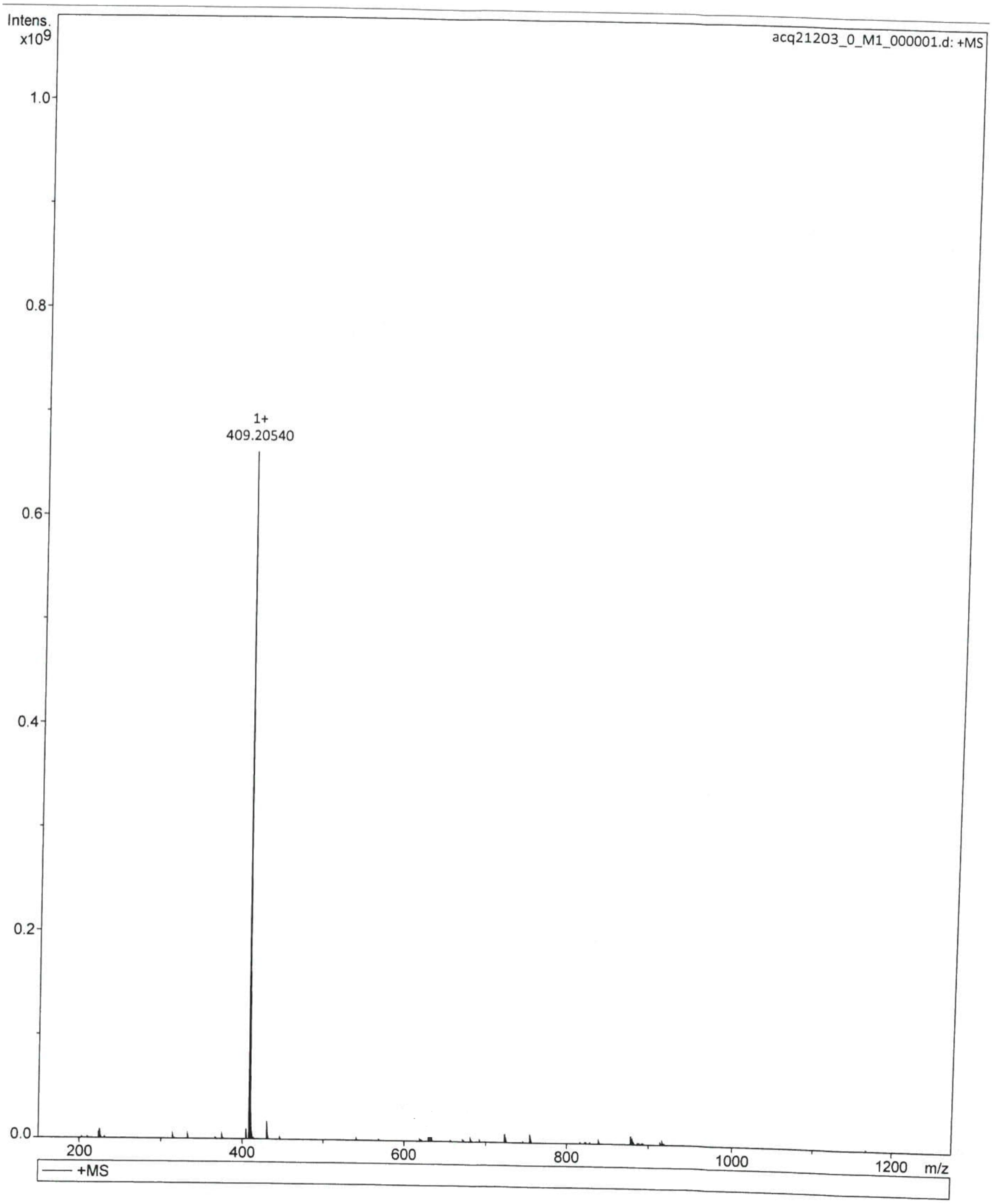

Figure S31. HRMS spectrum of compound **7e**

Generic Display Report

Analysis Info

Analysis Name	S:\fticr0\rod\analyser-rutine\acq21205_0_O1_000001.d	Acquisition Date	7/6/2022
Method	10800	Operator	
Sample Name		Instrument	solariX XR
Comment			

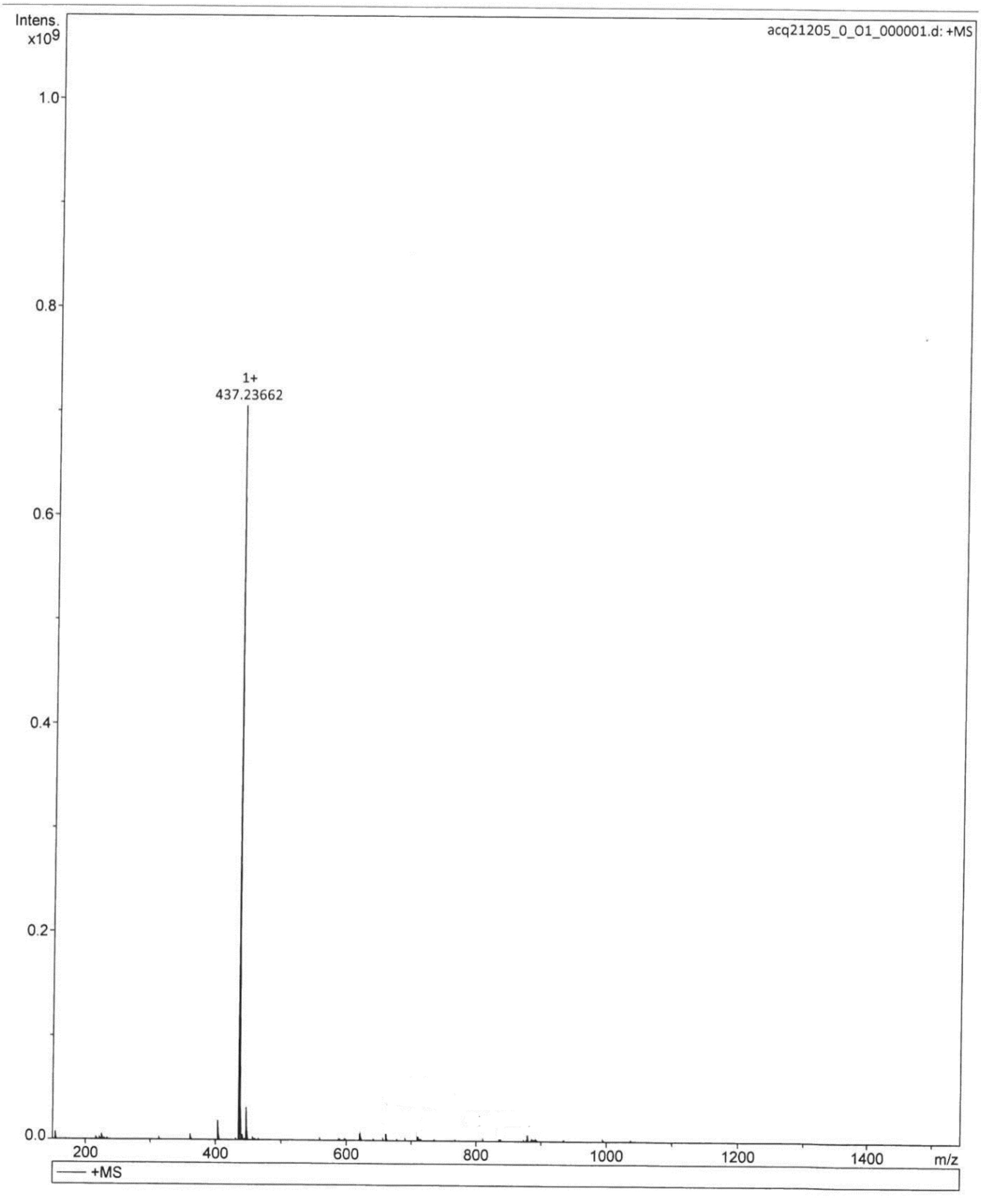

Figure S32. HRMS spectrum of compound **7f**
